# A Genome-Wide Association Study of Coleoptile Length in Different Chinese Wheat Landraces

**DOI:** 10.3389/fpls.2020.00677

**Published:** 2020-06-04

**Authors:** Jun Ma, Yu Lin, Si Tang, Shuonan Duan, Qing Wang, Fangkun Wu, Caixia Li, Xiaojun Jiang, Kunyu Zhou, Yaxi Liu

**Affiliations:** ^1^College of Agronomy and Biotechnology, China Agricultural University, Beijing, China; ^2^Triticeae Research Institute, Sichuan Agricultural University, Chengdu, China; ^3^State Key Laboratory of Crop Gene Exploration and Utilization in Southwest China, Chengdu, China

**Keywords:** candidate gene, coleoptile, genetic variation, *Triticum aestivum* L., validation

## Abstract

From the perspective of wheat yield improvement, the coleoptile is vital for successful crop establishment, and long coleoptile lengths (CLs) are preferred in wheat-growing regions where deep planting is practiced. To determine the genetic basis of CL, we performed a genome-wide association study on a set of 707 Chinese wheat landraces using 18,594 single-nucleotide polymorphisms and 38,678 diversity array technology sequencing markers. We accordingly detected a total of 29 significant markers [−log_10_^(P)^ > 4.76] distributed on chromosomes 2B, 2D, 3A, 4A, 5A, 6A, 6B, 6D, and 7B. Based on linkage disequilibrium decay distance, we identified a total of 17 quantitative trait loci associated with CL, among which *QCl.sicau-6B.2*, located at 508.17–509.26 Mb on chromosome 6B, was recognized as a novel major locus. We subsequently developed a high-resolution melt marker for *QCl.sicau-6B.2*, which was validated in an *F*_2__:__3_ population. Our findings provide important insights into the genetic mechanisms underlying coleoptile growth and could be applied to marker-assisted wheat selection.

## Introduction

Successful crop establishment is required for optimal yields and depends upon rapid shoot emergence and leaf area development. The coleoptile plays an important role in the establishment of wheat (*Triticum aestivum* L.), as it transports the young stem and first leaf from the embryo to the soil surface and protects the plumule as it moves through the soil layers. The coleoptile also determines the maximum depth at which wheat seeds can be sown. Approximately 37% of the wheat cropping area in developing countries consists of semi-arid/arid environments (Sinclair, 2011; Sadok, 2017), and compared to regions with higher precipitation levels, wheat is often planted deeper in these arid regions to ensure adequate soil moisture for germination (Schillinger et al., 1998; Mohan et al., 2013). With deep sowing, however, short coleoptiles might limit the ability of the seedling to push the first leaf through the soil surface, resulting in poor establishment. Therefore, wheat cultivars with long coleoptiles are preferable for deep planting.

Quantitative trait loci (QTLs) for coleoptile length (CL) have to date been mapped to wheat chromosomes 1A, 1B, 2B, 2D, 3A, 3B, 3D, 4A, 4B, 4D, 5A, 5B, 5D, 6A, 6B, 7A, and 7B, among which *Rht-B1b* (*Rht1*) and *Rht-D1b* (*Rht2*) are two major QTLs on chromosomes 4BS and 4DS, respectively (Rebetzke et al., 2007a, 2014; Spielmeyer et al., 2007; Yu and Bai, 2010; Singh et al., 2015; Li G. et al., 2016). Gibberellic acid (GA)-insensitive *Rht1* and *Rht2* are associated with reduced cell size and elongation and corresponding reductions in plant height (PH) and CL (Botwright et al., 2001, 2005). Since the advent of the Green Revolution in China, the dwarfing genes *Rht1* and *Rht2* derived from the cultivar Norin 10 have been used worldwide for dwarf wheat breeding (Worland and Sayers, 1995). Nevertheless, these have hindered wheat breeding for increased CL and reduced PH. In contrast, *Rht4*, *Rht5*, *Rht7*, *Rht8*, *Rht9*, *Rht12*, *Rht13*, and *Rht14* contribute to reducing wheat shoot height but do not affect the CL (Ellis et al., 2004; Botwright et al., 2005), and thus, these genes enable the breeding of dwarf cultivars with long coleoptiles. Moreover, prior to the Green Revolution, wheat landraces had been widely cultivated on a global scale. In China, there are more than 13,000 wheat landraces with relatively high PH (>110 cm) (Dong and Zheng, 2000). Thus, it is reasonable to speculate that the identification of QTLs controlling CL but not influencing PH in Chinese landraces might facilitate the breeding of dwarf cultivars with long coleoptiles.

A genome-wide association study (GWAS) is a powerful approach that can be used to facilitate the identification of QTLs encoding complex traits in plants. Unlike biparental mapping, GWAS exploits the advantages of phenotypic variation and historical recombination in natural populations. It can also overcome the limitations of biparental populations and enable high-resolution mapping (Nordborg and Weigel, 2008; Zhu et al., 2008). To date, GWAS has been widely used to identify QTLs in plants including *Arabidopsis* (Verslues et al., 2014; Bac-Molenaar et al., 2015), *Aegilops tauschii* (Liu et al., 2015a, b; Qin et al., 2016; Arora et al., 2017), and maize (Lu et al., 2012; Wen et al., 2014; Yang et al., 2014). In wheat, this technique has also been used to identify QTLs for agronomic traits (Liu Y. et al., 2017; Ogbonnaya et al., 2017), grain yield (Sukumaran et al., 2015; Sehgal et al., 2017), abiotic stress response (Mwadzingeni et al., 2017; Valluru et al., 2017; Lin et al., 2019), disease resistance (Jiang et al., 2015; Maccaferri et al., 2015), and quality-related characteristics (Lin et al., 2017; Liu J. et al., 2018). To date, however, only a single GWAS has been reported for the wheat coleoptile, in which four of eight potentially novel loci were identified for CL (Li G. et al., 2016). Accordingly, performing a GWAS for CL and growth might assist in the identification of novel loci and the development of new wheat varieties with the desired traits. In the present study, we collected 707 wheat landraces from 10 Chinese agro-ecological zones and used these to evaluate CL, in addition to conducting a GWAS on 57,272 markers to identify marker–trait associations and candidate genes.

## Materials and Methods

### Plant Material

In the present study, we used a core collection of 707 wheat landrace accessions, which were acquired from the following 10 agro-ecological zones in China: the Northern Winter Wheat Zone (NW), the Yellow and Huai River Valleys Facultative Wheat Zone (Y&H), the Middle and Low Yangtze Valleys Autumn-sown Spring Wheat Zone (YTS), the Southern Autumn-sown Spring Wheat Zone (SAS), the Southwestern Autumn-sown Spring Wheat Zone (SWAS), the Northeastern Spring Wheat Zone (NES), the Northern Spring Wheat Zone (NS), the Northwestern Spring Wheat Zone (NWS), the Qinghai–Tibetan Plateau Spring-Winter Wheat Zone (Q&T), and the Xinjiang Winter-Spring Wheat Zone (XJ). Detailed information on these 707 accessions is listed in Supplementary Table S1. As a validation population, we used an F_2__:__3_ population derived from the landraces Niqiuzhuan (NQZ) and Huolishao (HLS), which were obtained from an association mapping population and originated in zones III and I, respectively, with the former having a longer coleoptile than the latter.

### Phenotype Evaluation

The evaluation of CL was performed using a blotting-paper germination protocol (Li G. et al., 2016). A completely randomized block experimental design with three replications was used, and for each replication, 30 full, uniformly sized, and undamaged seeds from each accession were evaluated. In brief, two soaked filter papers were placed on wax paper, and the seeds for each accession were then placed in a line on the filter paper. The filter paper and wax paper were subsequently loosely rolled, secured with a rubber band, and arranged vertically in a plastic box. The plastic box was covered with black bags to exclude light and placed in refrigerator at a constant temperature of 4°C for 1 day to break dormancy. Thereafter, the box was transferred to a completely darkened growth chamber incubator at 15°C for 7 days, followed by 7 days at 20°C. Finally, the CLs of the 30 seedlings per accession were measured using a ruler as the distance from the scutellum to the tip of the coleoptile.

### Statistical Analysis

Means were determined for the 30 CLs from each independent replicate. To eliminate potential environmental effects, best linear unbiased prediction (BLUP) analyses were conducted among three replicates (Piepho et al., 2008). The means of three replicates (MR1, MR2, and MR3) and the BLUP were used for further analyses. Descriptive statistics and Pearson’s correlation coefficients were calculated for the means of each trait in all accessions using IBM SPSS Statistics for Windows v. 20.0 (IBM Corp., Armonk, NY, United States). Data on eight agronomic traits, namely, flowering date (FD), flag leaf length, flag leaf width, peduncle length (PDL), PH, seedling habits (SH), spike length, and tiller number were obtained from a previously published study (Liu Y. et al., 2017). The BLUP values of these eight traits were used to examine the correlations between plant morphology and CL. Analysis of variance was performed using SAS v. 8.1 (SAS Institute Inc., Cary, NC, United States). Broad-sense heritability (*H*^2^) was evaluated in SAS using the formula *H* = VG/(VG + VE), where VG is the genetic variance and VE is the environmental variance (Smith et al., 1998).

### Genotyping and Population Structure Analyses

Genomic DNA was extracted from leaf samples of the 707 Chinese wheat landraces and the *F*_2__:__3_ population using the cetyltrimethylammonium bromide method. Genotype by sequence libraries were constructed in 96-plex via the two-enzyme method (Poland et al., 2012) and sequenced using an Illumina HiSeq 2500 system. Single-nucleotide polymorphism (SNP) calling was performed using the TASSEL pipeline (Glaubitz et al., 2014). SNPs were assigned based on the Chinese Spring sequence [Reference sequence (RefSeq) v1.0; the International Wheat Genome Consortium (IWGSC)^1^ ] to obtain physical distances. SNPs were removed if they could not be assigned to any chromosome. Imputation accuracies were calculated using linkage disequilibrium (LD) *K*-number neighbor imputation (Money et al., 2015). SNPs with a minor allele frequency (MAF) < 0.05 and missing data > 20% were filtered, yielding a final total of 18,594 SNPs. In addition, we used a set of 38,678 diversity array technology sequencing (DArT-seq) markers that were mapped on the RefSeq v1.0 reference sequence generated in our previous study (Liu Y. et al., 2017).

Population structure was estimated using the STRUCTURE 2.3.4 program implementing model-based Bayesian cluster analysis (Pritchard et al., 2000). Population genetic clusters of *K* = 1–10 were evaluated using the admixture model with 10,000 replicates for burn-in and 10,000 replicates for Markov Chain Monte Carlo simulation. Five runs per *K* were performed, with the optimal *K*-value being determined using STRUCTURE HARVESTER (Earl and vonHoldt, 2012) based on the Evanno method (Evanno et al., 2005). The optimal alignment of the five replicates was determined using CLUMPP (Jakobsson and Rosenberg, 2007), and kinship among the 707 landraces was estimated using TASSEL 5 (Bradbury et al., 2007).

### Genome-Wide Association Study (GWAS)

A GWAS was conducted with TASSEL 5 using a compressed mixed linear model with optimal compression and variance component estimation based on previously determined population parameters (Bradbury et al., 2007; Zhang et al., 2010). A model with a *Q* matrix for the fixed effect and a kinship matrix for the random effect (Yu and Buckler, 2006) was used to identify marker–trait associations. Based on a Bonferroni correction (Yang et al., 2014), we set a threshold of −log_10_ (1/*n*, where *n* = the number of markers) to detect significant associations between DArT-seq markers and traits. For GWAS, we set a −log_10_ threshold of 4.76. Manhattan and quantile–quantile plots were generated using R v. 3.0.3^2^.

### Marker Development and QTL Validation

Based on the IWGSC RefSeq v. 1.0 sequence, extended sequences around significant markers were used to design primers using Primer-BLAST in the National Center for Biotechnology Information^3^ database. Sequences of the parental landraces NQZ and HLS were compared, and we searched for SNPs using DNAMAN v. 6.0 (Lynnon BioSoft; Vaudreuil-Dorion, Quebec, Canada). Single-base differences were identified via high-resolution melt marker (HRM) analysis (Wittwer et al., 2003; Wang et al., 2016; Zhou et al., 2016; Liu Y. et al., 2018). The SNPs were converted into HRM markers to track the identified loci by quantitative real-time polymerase chain reaction (qRT-PCR). The HRM markers were designed using Beacon Designer v. 7.9 and evaluated using Oligo v. 6.0 (Zhang and Gao, 2004). The optimal parameters for primer design and the qRT-PCR amplification reactions and the HRM marker polymorphisms, specificities, and sensitivities were determined based on previously described procedures (Wang et al., 2016; Zhou et al., 2016; Liu Y. et al., 2018).

For the development of HRM markers, the CLs of the homozygous NQZ/HLS lines were used to validate significant loci. Genotypes with homozygous alleles from NQZ were designated “AA” and those with homozygous alleles from HLS were identified as “aa.” Student’s *t*-tests were used to identify the differences in CL between these two classes and the loci effects in the validation population were calculated.

### Identification of Candidate Genes

The significant loci were used to identify candidate genes based on IWGSC RefSeq Annotation v. 1.0. The genes in the two aforementioned genomic regions were annotated using KOBAS v. 3.0 at *P* < 10^–5^ with *Arabidopsis thaliana* and *Oryza sativa* as background species (Wu et al., 2006). Identification of the candidate genes for CL was based on functional annotation.

## Results

### Phenotypic Analysis

**FIGURE 1:**
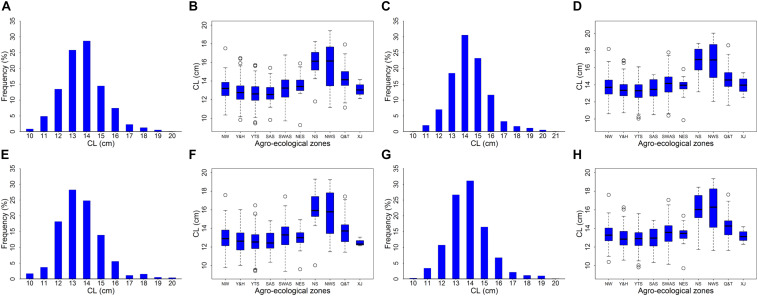
Distribution histograph and box plots for coleoptile length (CL). Panels **(A,C,E,G)** was distribution histograph for MR1, MR2, MR3, and BLUP, respectively. Panels **(B,D,F,H)** was box plots in MR1, MR2, MR3, and BLUP, respectively. NW, Northern Winter Wheat Zone; Y&H, Yellow and Huai River Valleys Facultative Wheat Zone; YTS, Middle and Low Yangtze Valleys Autumn-Sown Spring Wheat Zone; SAS, Southern Autumn-Sown Spring Wheat Zone; SWAS, Southwestern Autumn-Sown Spring Wheat Zone; NES, Northeastern Spring Wheat Zone; NS, Northern Spring Wheat Zone; NWS, Northwestern Spring Wheat Zone; Q&T, Qinghai-Tibetan Plateau Spring-Winter Wheat Zone; and XJ, Xinjiang Winter-Spring Wheat Zone.

We measured the CLs of 697 landraces and observed significant variation (*P* < 0.001) among the different genotypes and replicates (Table 1). Among these landraces, CL values ranged from 9.26 to 19.40 cm, 9.87 to 20.06 cm, 9.38 to 19.30 cm, and 9.72 to 19.34 cm for MR1, MR2, MR3, and BLUP, respectively (Figure 1 and Table 2). The coefficient of variation ranged from 10.76% to 11.86%, and the *H*^2^ of CL was 95.81%, thereby indicating high heritability (Table 2). Among the 10 assessed agro-ecological zones, we found that the CL in the NWS and Q&T zones was higher than that in other zones (Figure 1).

**TABLE 1:** Analysis of variance for coleoptile length.

**Variables**	**Degree of freedom**	**Type III sum of squares**	**Mean squares**	***F*-Value**	**Significance**
Genotype	696	138958.98	199.65	320.63	***
Repetition	2	7026.53	3513.26	5642.09	***
Genotype × Repetition	1392	9971.75	7.16	11.50	***

***Significant at the 0.001 probability level.

**TABLE 2:** Phenotypic variation and broad-sense heritability (*H*^2^) of coleoptile length.

**Variables**	**Minimum**	**Maximum**	**Mean**	***SD***^†^	**CV (%)**	***H*^2^ (%)**
MR1	9.26	19.40	13.22	1.53	11.57	
MR2	9.87	20.06	13.85	1.54	11.12	
MR3	9.38	19.30	13.07	1.55	11.86	
BLUP	9.72	19.34	13.38	1.44	10.76	95.81

*^†^SD, standard deviation; CV, coefficient of variation; H^2^, broad-sense heritability; MR1, means of coleoptile length in repetition 1; MR2, means of coleoptile length in repetition 2; MR3, means of coleoptile length in repetition 3; BLUP, best linear unbiased predictions.*

### Correlations Between CLs and Agronomic Traits

Correlation analyses performed for associations among CL measurements in the three replicates and BLUP revealed high correlations, with correlation coefficients ranging from 0.889 (between MR1 and MR3) to 0.970 (between MR2 and BLUP) (Table 3). To determine whether the CL of landraces is associated with PH and other characteristics, we performed correlation analyses between CL and each of the assessed agronomic traits. The correlation coefficients ranged from 0.008 (between MR2 and PH) to 0.235 (between MR3 and FD). The correlation coefficients ranged from 0.012 to 0.227 (Table 3). With the exception of PDL and PH, significantly positive correlations (*P* < 0.05 or *P* < 0.01) were observed between CL and all other agronomic traits. We found that CL was significantly negatively correlated (*P* < 0.01) with PDL. No significant correlation was observed between PH and CL. Thus, we could ascertain that certain QTLs or genes, which remain to be identified, are associated with CL but not with PH.

**TABLE 3:** Pearson correlations between coleoptile length and eight agronomic traits.

**Traits**	**MR1^†^**	**MR2**	**MR3**	**BLUP**	**FD**	**FLL**	**FLW**	**PDL**	**SH**	**SL**	**TN**	**PH**
MR1	1	0.914**	0.889**	0.967**	0.215**	0.145**	0.096*	−0.195**	0.201**	0.227**	0.219**	−0.072
MR2	0.914**	1	0.897**	0.970**	0.171**	0.139**	0.131**	−0.186**	0.215**	0.215**	0.156**	−0.063
MR3	0.889**	0.897**	1	0.962**	0.128**	0.145**	0.116**	−0.148**	0.209**	0.217**	0.137**	−0.012
BLUP	0.967**	0.970**	0.962**	1	0.177**	0.148**	0.119**	−0.182**	0.216**	0.227**	0.177**	−0.051

*Significant at the 0.05 probability level. **Significant at the 0.01 probability level. ^†^MR1, means of coleoptile length in repetition 1; MR2, means of coleoptile length in repetition 2; MR3, means of coleoptile length in repetition 3; BLUP, best linear unbiased predictions; FD, flowering date; FLL, flag leaf length; FLW, flag leaf width; PDL, peduncle length; SH, seedling habit; SL, spike length; TN, tiller number; PH; plant height.

Based on BLUP, we also analyzed the association between CL and PH using a quantile method in which the two extreme PH groups were segregated. Genotypes with PH values lower than the 10% quantile were classified as the shorter PH group (SPHG), whereas those with values higher than the 90% quantile were classified in the taller PH group (TPHG) (Valluru et al., 2017), and we accordingly found that the CLs of the SPHG sub-group were not significantly correlated with those of the TPHG sub-group (*r* = 0.007; *P* > 0.05), whereas PH was significantly positively correlated with CL in the SPHG sub-group (*r* = 0.310; *P* < 0.01).

### Molecular Markers, Population Structure, and Kinship

After filtering markers based on thresholds of MAF < 0.05 and missing data > 20%, we obtained a total of 57,272 polymorphic markers (18,594 SNP and 38,678 DArT-seq markers) for the 707 landraces, which were mapped on a physical reference map (Refseq v1.0). A total of 21,503, 25,365, and 10,404 markers were mapped onto the A, B, and D sub-genomes, respectively (Table 4). The marker density for the whole genome was 0.25 Mb/marker and 0.23, 0.20, and 0.38 Mb/marker for the A, B, and D sub-genomes, respectively. The number of markers per chromosome ranged from 476 for Chr. 4D to 4,885 for Chr. 2B, with Chr. 4D and Chr. 2B showing the lowest (1.07 Mb/marker) and highest (0.16 Mb/marker) marker densities, respectively. Based on the delta *K* model, the 707 landraces were divided into five sub-groups comprising 207, 185, 128, 105, and 82 landraces, respectively, as follows: sub-group 1 included most of the landraces from NW and Y&H and all of the landraces from XJ; sub-group 2 included most of the landraces from YTS and SAS; sub-group 3 included most of the landraces from SWAS and some of the landraces from Y&H and YTS; sub-group 4 included most of the landraces from IX and Q&T and some of the landraces from SWAS, NS, and NWS; sub-group 5 was a more diverse group that included most of the landraces from NEW and some of the landraces from Y&H, YTS, SWAS, and Q&T.

**TABLE 4:** The distribution of molecular marker in the whole genome.

**Chr**^†^	**Number of markers**				**Map length (Mb)**				**Marker density (Mb/marker)**			
	***A***	***B***	***D***	**Total**	***A***	***B***	***D***	**Total**	***A***	***B***	***D***	**Total**
1	2678	3543	1526	7747	593.45	689.28	495.25	1777.98	0.22	0.19	0.32	0.23
2	4048	4885	2678	11611	780.78	801.01	651.69	2233.48	0.19	0.16	0.24	0.19
3	2298	3825	1409	7532	750.76	829.98	615.46	2196.19	0.33	0.22	0.44	0.29
4	2995	1623	476	5094	744.54	673.45	509.85	1927.85	0.25	0.41	1.07	0.38
5	2715	3617	1152	7484	709.76	712.85	566.06	1988.66	0.26	0.20	0.49	0.27
6	2969	3878	1552	8399	618.00	720.95	473.52	1812.47	0.21	0.19	0.31	0.22
7	3800	3994	1611	9405	736.69	750.59	638.66	2125.94	0.19	0.19	0.40	0.23
All	21503	25365	10404	57272	4933.96	5178.11	3950.49	14062.56	0.23	0.20	0.38	0.25

*^†^Chr, chromosome.*

### Significant Markers Associated With CL

**FIGURE 2:**
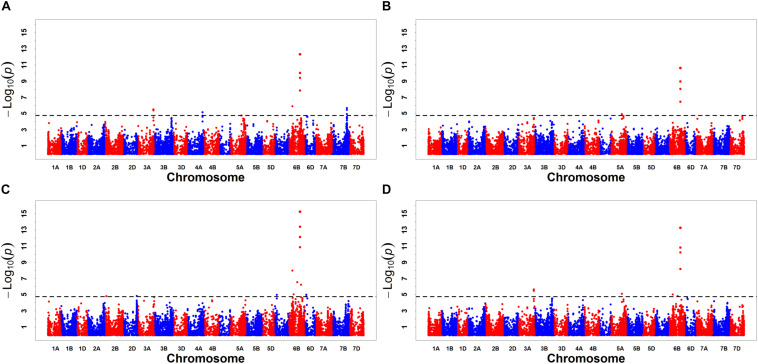
Manhattan plots for coleoptile length (CL). Panels **(A–D)** were the results detected for MR1, MR2, MR3, and BLUP, respectively.

A GWAS was performed on MR1, MR2, MR3, and BLUP, using a total of 57,272 DArT-seq markers, with a −log_10_^(*P*)^ value > 4.76 [−log_10_^(P)^ = −log_10_^(1/57,272)^] set as the threshold (Yang et al., 2014), to identify significant marker–trait associations. We accordingly detected a total of 29 significant markers for CL, with the amount of phenotypic variation explained (PVE) ranging from 2.70 to 10.64% (Figure 2 and Table 5). These markers were found to be distributed on chromosomes 2A, 2B, 2D, 3A, 3B, 4D, 5A, 6B, 7A, and 7D. Based on the LD decay distance, significant markers falling within a 5.98-Mb region were considered to represent a single QTL (Lin et al., 2019). In total, we detected 17 QTLs for CL, among which *QCl.sicau-6B.2*, located at 508.17–509.26 Mb on Chr. 6B, was considered a major locus and was detected in all three replicates and BLUP, with PVE values ranging from 3.10 to 8.17%.

**TABLE 5:** List of the significant markers for coleoptile length.

**QTL Name**	**Marker**	**Chr^†^**	**Position (Mb)**	**−LOG_10_^(P)^**				**PVE (%)**			
				**MR1**	**MR2**	**MR3**	**BLUP**	**MR1**	**MR2**	**MR3**	**BLUP**
*QCl.sicau-2A*	B32929	2A	764.66	–	6.03	5.18	6.07	–	4.10	3.57	4.17
	SNP3882	2A	764.66	6.43	6.32	6.39	7.36	3.75	3.68	3.74	4.36
*QCl.sicau-2B.1*	A30893	2B	39.41	4.93	–	–	–	2.80	–	–	–
*QCl.sicau-2B.2*	A30968	2B	47.85	–	–	4.96	–	–	–	2.97	–
*QCl.sicau-2D*	SNP6484	2D	651.77	–	5.23	–	5.01	–	2.96	–	2.82
*QCl.sicau-3A.1*	A48978	3A	404.69	–	5.20	–	–	–	3.13	–	–
	SNP6783	3A	404.69	–	4.87	–	–	–	2.73	–	–
*QCl.sicau-3A.2*	SNP6979	3A	700.22	5.28	–	–	5.13	3.00	–	–	2.90
	B11794	3A	700.22	5.34	–	–	5.16	3.03	–	–	2.92
	B11790	3A	700.22	5.29	–	–	5.13	3.00	–	–	2.90
	B11795	3A	700.22	5.26	–	–	5.11	2.99	–	–	2.90
*QCl.sicau-3B.1*	B10892	3B	755.81	5.23	5.20	–	5.39	3.08	3.07	–	3.19
	B10891	3B	755.81	4.85	4.82	–	5.00	2.83	2.82	–	2.94
	SNP8235	3B	755.81	5.44	5.34	–	5.58	3.10	3.04	–	3.19
*QCl.sicau-3B.2*	SNP8294	3B	770.47	5.32	5.67	–	5.76	3.02	3.25	–	3.31
*QCl.sicau-3B.3*	A11280	3B	813.01	–	–	4.80	–	–	–	2.84	–
*QCl.sicau-4D.1*	A13215	4D	319.09	–	–	4.92	–	–	–	2.99	–
*QCl.sicau-4D.2*	A39373	4D	498.96	–	5.24	–	–	–	3.02	–	–
*QCl.sicau-5A*	SNP11051	5A	564.56	–	4.92	–	4.89	–	2.76	–	2.75
	B14507	5A	564.56	5.02	5.17	–	5.13	2.86	2.94	–	2.92
	SNP11058	5A	566.55	–	4.92	–	4.89	–	2.76	–	2.75
*QCl.sicau-6B.1*	A48008	6B	168.77	5.85	–	5.00	5.50	3.52	–	3.04	3.32
*QCl.sicau-6B.2*	B18505	6B	508.17	10.53	9.30	12.02	11.49	6.93	6.07	8.17	7.67
	A17510	6B	508.17	6.44	5.36	6.19	6.42	3.85	3.10	3.71	3.82
	SNP14693	6B	508.17	7.54	6.54	7.74	7.75	4.48	3.82	4.63	4.62
	B18506	6B	509.26	7.97	7.94	10.19	9.33	5.08	5.09	6.80	6.09
*QCl.sicau-6B.3*	SNP14819	6B	628.06	–	–	5.07	–	–	–	2.88	–
*QCl.sicau-7A*	A19567	7A	536.82	–	–	4.77	–	–	–	2.76	–
*QCl.sicau-7D*	A888	7D	23.52	–	–	–	4.79	–	–	–	2.90

*^†^Chr, chromosome; PVE, phenotypic variation explained; MR2, means of coleoptile length in repetition 2; MR3, means of coleoptile length in repetition 3; BLUP, best linear unbiased predictions.*

### Development of an HRM Marker to Validate *QCl.sicau-6B.2*

**FIGURE 3:**
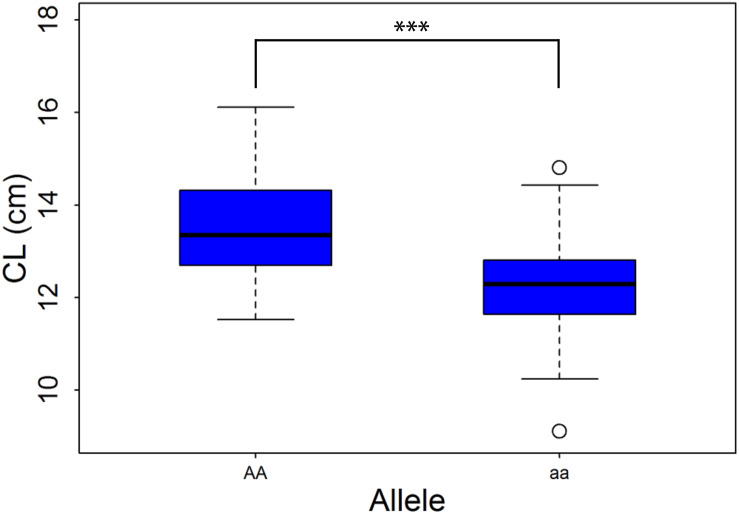
Box plots of coleoptile length (CL) in a *F*_2:3_ population. “AA” and “aa” represents homozygous alleles from D519 and D55, respectively. ***Significant at the 0.001 probability level.

A newly designed primer, HRM, was used to track *QTL-6B-2*, the information on which is presented in Supplementary Table S2. This marker was polymorphic in the *F*_2__:__3_ population, in which we detected 103 individuals with the homozygous “AA” genotype and 106 with the homozygous “aa” genotype. We found that the CL of the homozygous “AA” lines ranged from 11.53 to 16.11 cm, whereas that of the homozygous “aa” lines ranged from 9.11 to 14.81 cm (Figure 3). Furthermore, the average CL of NQZ landrace lines with homozygous genes was significantly greater than that of HLS lines with homozygous genes, and the effect was 9.48%.

### Predicted Genes Located in the Genomic Region of *QCl.sicau-6B.2*

The major QTL *QCl.sicau-6B.2* encompassed four significant markers, which were distributed over a 1.09-Mb region on chromosome 6B (Chr. 6B: 508.17–509.26 Mb) that contained 15 predicted genes (Supplementary Table S3). A gene ontology analysis indicated three genes with no specific annotation. Twelve of these genes were found to be homologous to genes occurring in *Arabidopsis*, including the four previously reported genes *G3Pp4* (Kawai et al., 2014), *WRKY12* (Wang et al., 2010; Sanchez et al., 2012; Li W. et al., 2016), *CYCP4;1* (Acosta et al., 2004), and *petN* (Sato et al., 1999), whereas 13 of the predicted genes were found to have rice homologs, including the three reported genes *P0491E01.9* (Kikuchi et al., 2003), *OrsajM_p08* (Hiratsuka et al., 1989), and ycf6 (Hiratsuka et al., 1989; Boeckmann et al., 2003).

## Discussion

We detected large phenotypic variations in the CL of 697 Chinese wheat landraces, with values ranging from 9.26 to 20.06 cm and a mean value of ∼13 cm. Consistently, based on an analysis of a worldwide collection of 893 wheat accessions, Li G. et al. (2016) obtained a mean CL of 13.33 cm. Furthermore, we found that landraces tend to have longer CLs than those previously determined for breeding lines or cultivars (Li G. et al., 2016), whereas in comparison, the CLs of an RIL population derived from a cross between the Chinese landrace Wangshuibai and the United States cultivar Wheaton ranged from 7.7 to 15.9 cm (Yu and Bai, 2010). Moreover, consistent with the findings of the present study, high correlations among experiments/repetitions and high heritabilities were detected in this RIL population and the aforementioned 893 worldwide wheat accessions (Yu and Bai, 2010; Li G. et al., 2016). Such high phenotypic variation and heritability was conducive to obtaining a high resolution for CL using GWAS.

The LD decay distance for Chinese wheat landraces was 5.98 Mb for the whole genome and 5.83, 5.28, and 6.00 Mb for A, B, and D sub-genomes, respectively (Lin et al., 2019), indicating that a large number of markers is required to achieve high resolution. We obtained a combined total of 57,272 SNP and DArT-seq markers with a marker density of 0.25 Mb/marker for the whole panel and sufficient SNP density for high-resolution mapping. Comparatively, Li G. et al. (2016) obtained an LD decay distance of 10 cM for the whole genome among the set of 893 global wheat accessions they examined and used 4,716 SNPs for further GWAS of CL. Previous studies on wheat have obtained LD decay values ranging from 1.5 to 15 cM, and found that the LD of the D sub-genome was higher than that of the A and B sub-genomes (Chao et al., 2010; Hao et al., 2011; Sukumaran et al., 2015; Li G. et al., 2016; Rahimi et al., 2019), which is consistent with the findings of the present study. We also found that the diversity panel could be divided into three sub-groups, which were largely consistent with geographical origins. Similarly, previous studies on wheat have reported that the characterization of sub-groups divided by Bayesian cluster analysis was largely consistent with geographical origins and pedigrees (Sukumaran et al., 2015; Li G. et al., 2016; Liu J. et al., 2017). In the present study, we considered population structure as a fixed-effect to reduce false positive errors and adopted MLM adjusted by population structure and kinship implemented in TASSEL v5.0 for association analysis.

Based on GWAS performed on 57,272 polymorphic markers associated with wheat CL, we detected a total of 29 markers that were significantly associated with this trait (Figure 2 and Table 5). The QTLs we thus identified for CL are comparable with those previously identified. Among these, the locus *QCl.sicau-6B.2*, which was associated with four significant markers (*B18505* and *B18506*, *SNP14693*, and *A17510*) in a 1.09-Mb genomic region (508.17–509.26 Mb) on Chr. 6B, was found to have the strongest effect on CL. Previous studies have reported 87 loci associated with CL (Supplementary Table S4), three of which are located on chromosome 6B (Rebetzke et al., 2007a, 2014; Singh et al., 2015). The markers closest to these QTLs were *barc178*, *gwm219*, and *P35/M39-9*, the first two of which are located at 664.63 and 674.84 Mb, respectively, and thus relatively remote from the loci identified in the present study. However, the *P35/M39-9* probe sequence was not published and cannot be compared based on the Chinese Spring physical map. According to Rebetzke et al. (2007a), the QTL interval of *P35/M39-9* was close to that of *barc178* and *gwm219* on their map. Using a MAGIC population, the two significant SNPs *wsnp_Ex_rep_c71537_70252046* and *wsnp_Ex_rep_c70767_69655253* were detected on chromosome 6B at 690.73 and 692.78 Mb, respectively (Rebetzke et al., 2014). Again, however, these are located at some distance from the loci detected in the present study. The QTL *qCL.6B.1* (with flanking markers *gpw1017* and *barc79*) has previously been mapped to chromosome 6B for CL in an RIL population (Singh et al., 2015). The marker *gpw1017* was not uniquely located on the Chinese Spring physical map, and thus, we compared the marker *barc79* located at 582.38 Mb with the locus identified in the present study. The distance between *barc79* and our locus was >70 Mb, thereby suggesting a novel locus for CL on chromosome 6B. We subsequently designed an HRM marker for this locus, which was validated in an *F*_2__:__3_ population, with an effect of 9.48% for CL. The gene *TraesCS6B01G282600*, located at 508.17–509.26 Mb on Chr. 6B, is a homolog of *CYCP4;1* (cyclin p4;1), a gene associated with cell division and differentiation that is strongly expressed in *Arabidopsis* hypocotyls (Acosta et al., 2004). Accordingly, we speculate that *TraesCS6B01G282600* might be a candidate gene associated with coleoptile traits.

In the present study, we found that with the exception of PH, there were significant correlations between CL and all other assessed agronomic traits (Table 3). However, we detected a significantly positive correlation between PH and CL in the SPHG sub-group (*r* = 0.310, *P* < 0.01), but not in the TPHG sub-group (*P* > 0.05), thereby indicating that CL is independent of PH among the TPHG landraces, which is consistent with the findings of previous studies. For example, in a comparison of nine semi-dwarf and seven tall wheat genotypes sown at depths, Rebetzke et al. (2007b) detected no significant correlation between CL and PH among the tall wheat varieties (Rebetzke et al., 2007b). In contrast, a positive correlation has been observed between PH and CL in wheat bearing the GA-insensitive dwarfing genes *Rht1* and *Rht2* (Rebetzke et al., 2007b; Liatukas and Ruzgas, 2011), which have the effect of suppressing CL and PH (Ellis et al., 2004). In the present study, we identified a total of 229 significant markers for the eight assessed agronomic traits based on GWAS (Supplementary Table S5). Interestingly, a genomic region for SH located at 647.97–650.68 Mb on Chr. 2D and a significant marker (SNP8280) for SH at 767.06 Mb on Chr. 3B were found to overlap with *QCl.sicau-2D* and *QCl.sicau-3B.2*, respectively. Furthermore, a significant marker (*A17437*) for PH located at 172.58 Mb on Chr. 6B was found to overlap with *QCl.sicau-6B.1*. Since the advent of the Green Revolution, *Rht1* and *Rht2* derived from the Norin 10 cultivar have been widely incorporated into modern wheat cultivars throughout the world (Worland and Sayers, 1995), as relatively shorter varieties with longer coleoptiles are preferred in wheat breeding. However, it is generally difficult to breed semi-dwarf wheat with long coleoptiles. Nevertheless, *Rht* 4, 5, 7, 8, 9, 12, 13, and 14 have been demonstrated to reduce PH in wheat without affecting the CL (Ellis et al., 2004; Botwright et al., 2005), and accordingly, these genes might facilitate the breeding of dwarf cultivars with long coleoptiles. In the present study, we screened a large number of Chinese wheat landraces in our attempt to identify loci for CL, and given the relatively high PH in Chinese landraces, we identified three loci associated with PH on chromosomes 2B, 6B, and 7D, only one of which was found to overlap with the loci detected for CL. This finding suggests that most of the landraces we examined do not carry the GA-insensitive dwarfing genes *Rht1* and *Rht2*. Thus, the identification of QTLs for CL in tall wheat varieties might enable breeding for coleoptile-related traits, and our newly identified locus combined with *Rht 4*, *5*, *7*, *8*, *9*, *12*, *13*, or *14* could be used to breed dwarf cultivars with long coleoptiles.

## Conclusion

The coleoptile of wheat is essential for the successful establishment of this crop. To identify the genetic basis of CL, we performed a GWAS based on 57,272 SNP and DArT-seq markers for this trait, and accordingly identified a total of 29 significant markers. Among these, a novel locus was identified on chromosome 6B and validated in an *F*_2__:__3_ population. Based on annotation, we suggest that *TraesCS6B01G282600*, which is associated with cell division and differentiation, is a candidate gene for this locus. In our future research, we intend to use the validated locus in molecular-assisted breeding to develop wheat cultivars with long coleoptiles. The candidate gene will also be validated by qRT-PCR and applied to transgenic tests.

## Data Availability Statement

The datasets GENERATED for this study can be found in the EVA (https://www.ebi.ac.uk/eva/); Project: PRJEB38030 and Analyses: ERZ1309082.

## Author Contributions

JM and YuL performed the phenotypic evaluation, conducted data analysis, and drafted the manuscript. ST, SD, QW, FW, and CL performed the phenotypic evaluation and helped with data analysis. XJ helped to draft the manuscript. KZ performed part of the statistical analyses. YaL designed and coordinated the study and revised the manuscript. All authors read and approved the final draft of the manuscript.

## Conflict of Interest

The authors declare that the research was conducted in the absence of any commercial or financial relationships that could be construed as a potential conflict of interest.

## Footnotes

1 http://www.wheatgenome.org/

2 http://www.r-project.org/

3 https://www.ncbi.nlm.nih.gov/tools/primer-blast/index.cgi?LINK_LOC=BlastHome
