# Supplementary material for: A Genome-Wide Association Study of Coleoptile Length in Different Chinese Wheat Landraces
Source: Front Plant Sci. 2020 Jun 4;11:677. doi: 10.3389/fpls.2020.00677 (PMC7287122; doi:10.3389/fpls.2020.00677)
Supplement: Supplementary file 1 [file Data_Sheet_1.PDF]

Supplemental Table S1 Information of the 707 wheat accessions assessed in the present study.

Accession No.	Landrace name	Origin	Geographic distribution†	Atitude (m)	East longitude (°)	Northern Latitude (°)	BLUP of 8 agronomic traits‡							
							FD	FLL	FLW	PDL	SH	SL	TN	PH
AS660683	Chongyanghongmai	Hubei	YTS	-	119.18	36.75	162.99	19.8	1.56	23.45	1.82	11.05	15.19	148.02
AS660689	Huangshuibai	Zhejiang	YTS	-	-	-	-	-	-	-	-	-	-	-
AS660739	Chaoanxiaomai	Guangdong	SAS	-	119.18	36.75	153.48	21.84	1.58	23.71	2.11	9.43	10.38	123.7
AS660789	Baituzitou	Shangdong	Y&H	-	-	-	173.45	20.41	1.14	24.3	1.38	9.59	14.6	136.8
AS660889	Kashibaipi	Xinjiang	XJ	58	119.18	36.43	161.72	21.84	1.79	22.43	2.11	12.86	13.6	135.39
AS660891	Chinese Spring	Sichuan	SWAS	65	116.22	40.22	157.29	24.21	1.6	29.17	2.26	10.06	13.77	143.19
AS661005	Baixiaomai	Beijing	NW	5	116.92	38.93	165.85	20.07	1.41	26.69	1.09	10.38	10.38	142.19
AS661006	Baimangmai	Beijing	NW	46	114.50	36.58	164.89	21.14	1.41	26.25	1.09	9.09	10.92	135.96
AS661007	Guanghulutou	Haidian, Beijing	NW	-	115.97	39.48	172.82	26.1	1.41	21.86	1.09	11.49	12.96	135.72
AS661009	Tumai	Tongxian, Beijing	NW	16	116.80	37.65	164.26	19.29	1.28	23.1	1.09	8.66	12.7	139.78
AS661010	Wuhuatou	Tongxian, Beijing	NW	750	111.78	37.25	168.38	17.44	1.21	18.5	1.09	10.23	11.52	141.62
AS661011	Hongmanghong	Shunyi, Beijing	NS	-	110.45	34.88	164.89	23.2	1.36	23.47	1.09	10.53	13.52	137.26
AS661012	Henanbai	Fangshanzhoukoudian, Beijing	NW	42	122.03	37.18	168.06	19.59	1.36	18.87	1.09	8.18	11.65	134.3
AS661013	Baimang	Tongxian, Beijing	NW	60	116.80	35.77	174.72	24.17	1.38	25.21	1.09	10.59	12.8	135.01
AS661016	Daqingmang	Junliangcheng, Tianjin	NW	28	120.20	36.03	166.48	18.86	1.32	23.47	1.09	9.65	12.28	135.51
AS661017	Hongmangbai	Jinghai, Tianjin	NW	66	117.52	36.72	164.89	19.32	1.41	25.75	1.09	10.16	12.31	143.73
AS661018	Baihulutou	Ninghe, Tianjin	NW	34	116.25	36.33	167.11	19.13	1.36	22.99	1.09	8.35	11.54	130.84
AS661019	Xiaobaimang	Wuyi, Hebei	Y&H	184	111.50	33.13	162.99	18.29	1.36	24.17	1.09	9.57	10.54	147.74
AS661021	Youzimai	Zaoqiang, Hebei	Y&H	42	115.07	33.38	165.53	19.34	1.49	24.04	1.09	8.3	12.12	135.02
AS661023	Wuhuatou	Xianxian, Hebei	Y&H	62	114.02	33.40	167.43	17.39	1.32	24.34	1.09	8.96	11.99	138.45
AS661024	Baipibai	Tangshan, Hebei	NW	217	111.50	33.30	164.26	21.91	1.41	24.47	1.09	7.75	13.57	131.01
AS661027	Xiaomaimang	Shulu, Hebei	Y&H	803	110.07	36.58	171.55	23.83	1.38	16.48	1.09	11.06	17.41	142.34
AS661028	Baimai39F	Zhengding, Hebei	NW	320	109.37	32.85	156.97	17.47	1.66	19.63	1.24	10.21	9.7	122.45
AS661030	Gongxianmai	Jinxian, Hebei	Y&H	800	109.50	35.25	166.8	20.64	1.32	22.45	1.09	9.49	12.05	139.94
AS661033	Yulinbai	Baixiang, Hebei	Y&H	445	129.00	42.55	161.09	17.97	1.34	18.65	1.09	7.15	11.34	115.8
AS661034	Daheshangtou	Handan, Hebei	Y&H	-	113.87	40.87	165.21	22.31	1.49	26.86	1.09	6.98	14.09	130.23
AS661035	Baike	Anci, Hebei	NW	2100	103.25	36.75	167.43	19.55	1.39	22.58	1.09	8.26	11.34	141.09
AS661037	Dabaimai	Xingtang, Hebei	Y&H	2312	97.55	39.80	165.21	22.86	1.36	24.82	1.38	9.37	12.57	145.1
AS661038	Dabailing	Xinle, Hebei	Y&H	2835	102.27	35.10	165.53	21.32	1.38	24.93	1.09	10.03	14.19	146.35
AS661039	Zijingbai	Zhengding, Hebei	NW	3	120.63	31.15	164.26	18.93	1.39	27.99	1.09	8.6	11.76	145.04
AS661041	Xiaobaipi	Jinxian, Hebei	Y&H	5	120.27	31.55	163.94	22.48	1.36	26.1	1.09	9.93	12.54	146.66
AS661042	Liulengmai	Jinxian, Hebei	Y&H	6	119.80	31.37	167.75	21.79	1.32	24.06	1.09	9.9	13.77	141.51
AS661046	Dongguangtou	Tangshan, Hebei	NW	9	118.83	32.35	-	-	-	-	-	-	-	-
AS661053	Baiqingtinghong	Baoding, Hebei	NW	5	120.23	32.08	170.28	21.93	1.32	25.86	1.09	10.72	12.76	138.63
AS661054	Daqitou	Baoding, Hebei	NW	4	119.83	34.00	170.92	24.21	1.52	22.23	1.09	9.25	12.02	136.6
AS661056	Tutoubai	Zhuoxian, Hebei	NW	33	118.75	34.52	164.58	18.03	1.21	22.49	1.09	9.83	12.34	140.77
AS661062	Baixiaomai	Cangxian, Hebei	Y&H	4	121.38	31.62	175.04	23.3	1.41	24.17	1.09	10.01	14.15	148.04
AS661065	Dabaimang	Qingxian, Hebei	NW	9	120.13	30.18	166.8	19.79	1.43	24.45	1.09	9.79	12.2	144.92
AS661066	Chunqiumaizi	Qingxian, Hebei	NW	-	119.20	29.37	166.48	19.33	1.24	22.14	1.09	7.98	15.87	131.99
AS661067	Xiaohongmang	Qingxian, Hebei	NW	5	121.15	30.05	152.85	18.15	2.05	15.11	2.55	9.67	9.84	103.74
AS661069	Hongtutou	Mengcun, Hebei	Y&H	60	119.92	28.45	168.38	19.31	1.67	18.11	1.38	10.65	13.25	122.27
AS661071	Hongmangbai	Jiaohe, Hebei	Y&H	95	118.52	28.88	166.48	21.98	1.38	30.04	1.09	9.82	12.89	145.01
AS661072	Hongmangmai	Zaoqiang, Hebei	Y&H	151	120.07	28.65	168.06	21.42	1.45	20.04	1.09	10.28	13.89	147.77
AS661073	Baimangmai	Jixian, Hebei	Y&H	598	113.68	25.57	165.21	21.2	1.41	27.99	1.09	9.96	12.67	137.35
AS661074	Guchengxiaomai	Gucheng, Hebei	Y&H	4	113.20	23.37	165.85	22.53	1.3	27.45	1.09	10.3	11.89	146.82
AS661075	Gaochan1Hao	Ningjin, Hebei	Y&H	635	108.13	32.08	168.06	21.2	1.38	21.21	1.09	8.52	11.21	130.58
AS661076	Honghulubaimai	Xingtai, Hebei	Y&H	338	104.52	28.43	164.58	19.96	1.24	24.3	1.09	9.95	10.67	140.65
AS661078	Dahongmang	Xingtai, Hebei	Y&H	1788	102.25	26.67	162.99	20.02	1.23	24.82	1.09	9.72	13.36	141.58
AS661079	Baikebai	Ningjin, Hebei	Y&H	375	105.70	29.72	165.21	22.36	1.45	26.73	1.09	10.35	12.73	140.41
AS661080	Baihulutou	Guangzong, Hebei	Y&H	161	108.90	30.97	164.89	22.21	1.38	29.97	1.09	9.63	13.02	138.76
AS661081	Sumai	Xiaoyi, Shanxi	NW	293	106.28	30.35	161.41	19.51	1.52	22.3	1.67	12.09	11.15	134.24
AS661083	Heshangtou	Zuoquan, Shanxi	NW	310	105.07	29.58	164.58	23.88	1.47	22.89	1.09	9.7	12.72	124.7
AS661094	Dabaimai	Pingding, Shanxi	NW	667	107.88	28.52	172.82	20.63	1.36	14.82	1.09	8.86	15.02	130.64
AS661096	Tuxiaomai	Lingshi, Shanxi	NW	1450	105.22	24.20	167.11	20.5	1.24	23.49	1.09	8.67	15.12	139.31
AS661097	Xiaomangmai	Lishi, Shanxi	NW	140	127.42	46.12	166.48	21.96	1.41	23.65	1.09	8.03	11.86	147.01
AS661099	Dadongxiaomai	Fenyang, Shanxi	NW	15	119.38	25.72	162.68	19.76	1.34	25.3	1.09	9.36	12.22	147.8
AS661102	Baimangmai	Pingshun, Shanxi	NW	8	117.38	37.77	163.94	18.43	1.19	23.63	1.09	9	11.26	141.85
AS661103	Zhuganqing	Gaoping, Shanxi	NW	200	110.82	32.85	162.04	16.84	1.43	10.78	1.24	8.48	10.38	118.55
AS661104	Baimanghong	Gaoping, Shanxi	NW	-	114.28	29.88	167.43	20.91	1.38	24.15	1.09	9.61	13.96	131.89
AS661107	Baiguandong	Hongdong, Shanxi	NW	310	111.58	27.07	158.55	18.55	1.39	21.19	1.09	8.15	11.67	126.38
AS661108	Huolishao	Hongdong, Shanxi	NW	1025	105.62	25.95	164.89	18.38	1.21	25.15	1.09	8.31	12.47	141.29
AS661109	Mangmai	Hongdong, Shanxi	NW	1340	100.97	22.78	168.7	20.64	1.38	23.49	1.24	10.1	14.23	137.46
AS661110	Baixianmai	Anyi, Shanxi	NW	570	109.15	32.43	166.48	17.94	1.19	21.04	1.09	9.12	15.38	135.96
AS661111	Baimangmai	Wanrong, Shanxi	NW	423	108.78	34.60	163.31	20.89	1.32	24.15	1.38	9.18	10.38	136.06
AS661112	Youmangshangeda	Jieyu, Shanxi	NW	320	109.37	32.85	158.24	17.89	1.39	20.08	1.09	7.46	12.25	126.98
AS661114	Hongbi	Jieyu, Shanxi	NW	2100	103.25	36.75	161.41	20.3	1.41	24.36	1.09	10.11	9.99	134.71
AS661115	Yangzhongsanyuehuang	Linyi, Shanxi	NW	-	98.10	36.30	160.14	16.4	1.26	15.45	1.53	8.18	13.56	117.25
AS661116	Baishanmai	Linyi, Shanxi	NW	-	97.18	31.15	161.41	16.6	1.21	21.17	1.38	9.57	14.93	132.7
AS661117	Louguding	Jinan, Shandong	Y&H	-	91.95	27.98	160.14	14.21	1.34	18.06	1.53	9.7	8.99	117.59
AS661118	Qimai	Longkou, Shandong	Y&H	-	94.38	29.57	174.4	19.99	1.66	26.6	1.24	12.39	11.02	139.03
AS661119	Jinbaoyu	Yixian, Shandong	Y&H	-	91.37	29.67	168.06	17.32	1.32	26.06	1.09	10.09	13.44	147.83
AS661120	Banmangmai	Boshan, Shandong	Y&H	-	89.72	28.58	166.8	17.97	1.43	23.91	1.09	8.64	12.54	145.86
AS661122	Zijielumai	Jimo, Shandong	Y&H	-	87.07	28.62	168.06	18.82	1.39	24.84	1.09	9.16	10.52	146.33
AS661123	Bodongqing	Jimo, Shandong	Y&H	-	89.62	28.92	177.26	22.4	1.47	36.79	1.09	9.66	12.64	158.34
AS661124	Hongkangbai	Rongcheng, Shandong	Y&H	2255	100.85	27.30	175.99	24.6	1.34	23.41	1.09	10.15	14.22	146.45
AS661125	Biansui	Wendeng, Shandong	Y&H	-	91.78	29.22	176.94	22.49	1.38	31.36	1.09	10.97	13.47	145.65
AS661126	Hongmangxiaomai	Wendeng, Shandong	Y&H	-	91.67	29.05	167.11	17.9	1.54	22.84	1.09	9.66	11.02	148.58
AS661127	Baiguangtou	Mouping, Shandong	Y&H	30	121.03	36.78	176.94	22.34	1.39	28.41	1.09	9.87	13.18	140.47
AS661128	Laohongtuxiaomai	Haiyang, Shandong	Y&H	135	120.85	37.30	166.16	18.53	1.26	24.56	1.09	10.49	13.06	141.63
AS661129	Xiaogongxian	Qixia, Shandong	Y&H	-	-	-	175.04	22.36	1.32	26.91	1.09	8.89	18.11	144.91
AS661130	Hongmangmai	Weixian, Shandong	Y&H	-	-	-	164.58	20.69	1.47	27.1	1.09	9.04	13.54	148.87
AS661131	Xiaohongmang	Weixian, Shandong	Y&H	55	119.92	36.75	164.58	20.38	1.34	22.69	1.09	9.55	13.09	136.4
AS661132	Baishamai	Pingdong, Shandong	Y&H	-	-	-	174.4	21.2	1.56	26.75	1.24	10.93	13.15	146.17
AS661134	Xiaobiansui	Anqiu, Shandong	Y&H	60	116.80	35.77	165.21	20	1.54	20.23	1.24	8.12	11.54	135.86
AS661136	Baimai	Ningyang, Shandong	Y&H	24	116.65	39.90	162.36	18.86	1.3	20.93	1.09	8.83	14.1	143.73
AS661137	Baitutou	Ningyang, Shandong	Y&H	107	116.77	36.18	160.77	18.77	1.47	21.5	1.09	9.78	12.15	127.91
AS661138	Banjiemangdabaimai	Feicheng, Shandong	Y&H	48	116.45	35.93	161.09	17.54	1.38	23.73	1.09	9.71	9.89	131.07
AS661139	Mazhatoumai	Dongping, Shandong	Y&H	66	117.52	36.72	-	-	-	-	-	-	-	-
AS661140	Hongmanghongmai	Zhangqiu, Shandong	Y&H	9	118.12	37.15	161.72	22.1	1.47	24.69	1.09	10.82	13.06	146.58
AS661141	Xiaobaimangmai	Boxing, Shandong	Y&H	24	116.37	37.17	167.11	18.28	1.34	19.45	1.09	8.96	11.55	130.05
AS661143	Baihulutou	Dongxian, Shandong	Y&H	40	116.10	35.80	164.58	19.88	1.34	22.76	1.09	10.79	11.96	140.71
AS661145	Baikumai	Liangshan, Shandong	Y&H	36	116.30	35.07	169.02	17.66	1.49	17.65	1.09	9.22	14.77	139.1
AS661147	Hongchoumai	Jinxiang, Shandong	Y&H	39	117.07	36.68	170.28	18.26	1.39	20.63	1.09	9.87	13.83	140.91
AS661148	Bailouguding	Licheng, Shandong	Y&H	2	117.65	39.02	171.55	22.72	1.86	13.56	2.26	10.46	12.28	119.54
AS661149	Baikangyangmai	Qingdao, Shandong	Y&H	91	117.97	36.65	167.75	18.63	1.39	27.04	1.09	11.21	13.12	147.57
AS661150	Hongheshang	Zichuan, Shandong	Y&H	-	117.80	34.77	176.31	21.98	1.34	31.32	1.09	9.83	12.41	154.43
AS661151	Huomai	Yixian, Shandong	Y&H	-	116.32	37.43	161.09	18.05	1.15	24.43	1.09	8.04	12.02	133.97
AS661152	Yejiling	Deping, Shandong	Y&H	24	116.37	37.17	164.58	19.89	1.39	31.54	1.09	10.59	10.8	153.7
AS661153	Yazizui	Pingyuan, Shandong	Y&H	20	117.20	36.97	170.28	19.43	1.54	22.93	1.09	9.86	11.67	146.38
AS661156	Bimangbai	Jiyang, Shandong	Y&H	7	121.27	37.50	168.06	22.18	1.34	18.63	1.09	9.8	13.8	141.89
AS661159	Laiyangzhuang	Fushan, Shandong	Y&H	15	122.40	37.17	167.11	19.63	1.47	29.21	1.09	9.24	12.7	152.52
AS661160	Shidaosankemang	Rongcheng, Shandong	Y&H	26	120.45	36.38	169.02	22.41	1.64	28.17	1.09	10.06	11.25	136.85
AS661162	Xiaohongmangliangmai	Jimo, Shandong	Y&H	36	120.52	37.65	166.8	20.14	1.26	28.91	1.09	9.61	11.63	142.25
AS661163	Piaoxiao	Huangxian, Shandong	Y&H	-	-	-	173.77	21.27	1.34	21.52	1.09	10.87	14.43	134.42
AS661164	Baitumai	Zhangqiu, Shandong	Y&H	190	117.65	36.25	172.5	21.94	1.54	23.82	1.09	9.32	13.38	136.94
AS661165	Changmangbai	Laiwu, Shandong	Y&H	60	116.80	35.77	162.68	18.94	1.3	22.71	1.09	10.3	11.15	140.33
AS661166	Hongdongmai	Ningyang, Shandong	Y&H	48	116.45	35.93	173.45	20.92	1.34	26.28	1.09	9.93	14.36	138.59
AS661168	Jinmai	Dongping, Shandong	Y&H	48	116.45	35.93	164.58	19.13	1.3	29.64	1.09	8.76	11.57	151.46
AS661169	Luanrangxian	Dongping, Shandong	Y&H	48	116.45	35.93	165.53	20.09	1.54	24.8	1.09	9.67	15.44	142.7
AS661170	Dabaimang	Dongping, Shandong	Y&H	40	116.50	36.23	166.48	17.48	1.34	22.89	1.09	9.61	12.68	146.53
AS661171	Hongmangguozitou	Changqing, Shandong	Y&H	36	116.30	35.07	161.09	18.24	1.43	24.41	1.09	8.11	11.41	143
AS661173	Kaoshanhong	Jinxiang, Shandong	Y&H	43	116.47	35.72	165.21	20.7	1.36	27.32	1.09	9.6	14.96	137.91
AS661174	Yulincao	Wenshang, Shandong	Y&H	51	115.52	35.55	161.72	17.17	1.38	25.58	1.09	9.05	12.89	134.85
AS661176	Choumai	Juancheng, Shandong	Y&H	3	117.82	39.33	171.87	19.1	1.41	27.82	1.09	9.67	15.46	145.07
AS661179	Baimangyouzi	Neihuang, Henan	Y&H	83	113.38	35.33	159.82	17.64	1.38	19.78	1.09	7.41	13.22	126
AS661182	Baigedatou	Xiuwu, Henan	Y&H	268	112.43	34.65	161.72	18.75	1.39	21.17	1.09	6.33	12.22	122.76
AS661183	Dakoumai	Luoyang, Henan	Y&H	750	111.60	33.78	164.58	20.61	1.52	29.12	1.24	10.39	12.05	149.02
AS661184	Cantiaomai	Luanchuan, Henan	Y&H	200	112.42	34.42	161.72	18.37	1.52	23.91	1.82	10.15	11.21	151.86
AS661185	Yingsizhuang	Yichuan, Henan	Y&H	115	113.45	34.15	162.04	17.98	1.54	19.76	1.24	9.58	10.15	147.1
AS661187	Xiaozibai	Yuxian, Henan	Y&H	147	113.32	32.72	163.31	20.61	1.38	26.82	1.09	8.79	14.35	141.12
AS661188	Datouben	Miyang, Henan	Y&H	45	114.85	33.72	165.21	18.82	1.49	22	1.09	7.89	12.05	143.86
AS661190	Dongcaomai	Huaiyang, Henan	Y&H	120	112.53	32.98	162.68	19.19	1.23	21.82	1.09	8.34	15.83	143.56
AS661191	Kumai	Nanyang, Henan	Y&H	23	115.90	37.78	169.65	19.39	1.34	20.54	1.09	10.33	11.67	141.06
AS661194	Pushanbamai	Xichuan, Henan	Y&H	100	113.63	34.78	166.48	22.28	1.9	26.04	1.67	12.65	12.38	144.32
AS661197	Huangguaxian	Zhengzhou, Henan	Y&H	73	114.18	35.13	167.75	18.79	1.24	23.41	1.09	9.63	12.89	140.89
AS661198	Dabaimai	Yanjin, Henan	Y&H	73	114.18	35.13	165.21	17.59	1.23	24.84	1.09	9.55	12.7	138.22
AS661199	Baicaomai	Yanjin, Henan	Y&H	149	112.58	35.10	162.68	15.83	1.28	21.58	1.38	7.99	10.76	126.04
AS661200	Mazhamai	Jiyuan, Henan	Y&H	149	112.58	35.10	162.68	18.93	1.32	24.06	1.24	8.05	10.75	128.2

Supplemental Table S1 Information of the 707 wheat accessions assessed in the present study.

Accession No.	Landrace name	Origin	Geographic distribution[†]	Atitude (m)	East longitude (°)	Northern Latitude (°)	BLUP of 8 agronomic traits[‡]							
							FD	FLL	FLW	PDL	SH	SL	TN	PH
AS661201	Xiaofoshou	Jiyuan, Henan	Y&H	49	115.12	35.90	162.68	18.33	1.62	24.26	1.24	9.07	11.67	135.29
AS661202	Benyouzimai	Qingfeng, Henan	Y&H	400	113.38	34.53	161.09	16.36	1.32	18.82	1.38	8.2	8.94	125.17
AS661203	Baitiaoyu	Mixian, Henan	Y&H	49	115.80	34.45	161.72	18.52	1.54	29.25	1.09	10.83	12.18	141.88
AS661204	Benhulutou	Shangqiu, Henan	Y&H	62	115.13	34.65	165.53	16.37	1.39	21	1.09	8.44	11.49	128.5
AS661205	Wuhuatou	Minquan, Henan	Y&H	24	115.75	37.50	165.85	17.81	1.41	24.8	1.09	9.51	11.15	143.78
AS661206	Baimangbai	Shenqiu, Henan	Y&H	45	114.85	33.72	161.41	18.4	1.66	21.82	1.24	7.61	11.57	139.92
AS661207	Tutoumai	Huaiyang, Henan	Y&H	268	112.43	34.65	162.04	19.53	1.3	29.93	1.09	8.98	11.54	147.28
AS661208	Laochushanbao	Luoyang, Henan	Y&H	406	110.87	34.52	164.89	17.41	1.75	22.36	1.09	9.04	12.41	148.16
AS661209	Lingbao925	Lingbao, Henan	Y&H	397	111.18	34.77	162.68	18.66	1.45	26.69	1.09	8.68	11.15	141.26
AS661210	Gedatou	Shanxian, Henan	Y&H	397	111.18	34.77	162.36	18.8	1.54	20.69	1.09	9.28	10.76	135.95
AS661211	Sanyuehuang	Shanxian, Henan	Y&H	249	112.15	34.72	159.51	16.98	1.1	22.95	1.09	8.52	12.67	137.89
AS661212	Gedasuihongmai	Xinan, Henan	Y&H	95	114.05	32.80	162.99	17.67	1.54	21.15	1.09	7.51	9.73	130.1
AS661215	Baimai	Queshan, Henan	Y&H	95	114.05	32.80	163.31	23.43	1.43	28.1	1.09	9.5	17.82	128.01
AS661216	Baimangcao	Queshan, Henan	Y&H	65	114.02	33.15	161.72	19	1.3	24.21	1.09	10.09	12.75	148.4
AS661217	Dalihong	Suiping, Henan	Y&H	13	116.12	38.18	162.68	17.93	1.38	25.47	1.09	10.42	12.73	152.84
AS661219	Hongmanghong	Xiping, Henan	Y&H	73	114.27	33.27	166.16	20.93	1.34	26.36	1.24	10.02	14.48	149.46
AS661220	Youzitou	Shangcai, Henan	Y&H	73	114.27	33.27	165.21	18.55	1.54	23.15	1.24	7.42	11.21	146.42
AS661221	Zihuatou	Shangcai, Henan	Y&H	49	114.35	33.00	164.58	19.51	1.51	26.69	1.24	9.77	14.48	139.72
AS661222	Benmai	Runan, Henan	Y&H	46	114.98	32.73	162.68	17.45	1.26	25.8	1.09	9.97	14.77	151.48
AS661223	Baisuibai	Pingyu, Henan	Y&H	158	113.00	33.27	166.48	17.78	1.32	24.06	1.09	9.4	13.15	144.18
AS661224	Qumangmai	Fangcheng, Henan	Y&H	158	113.00	33.27	167.11	18.83	1.58	21.82	1.09	11.87	11.02	145.32
AS661225	Dongmangmai	Fangcheng, Henan	Y&H	92	112.40	32.52	156.97	17.36	1.43	18.52	1.09	10.6	11.44	134.47
AS661226	Xiaobaimai	Xinye, Henan	YTS	92	112.40	32.52	162.04	18.93	1.36	25.28	1.09	10.37	12.7	144.69
AS661227	Liangganbai	Xinye, Henan	YTS	184	111.50	33.13	168.7	20.51	1.47	18.5	1.24	11.59	13.8	143.41
AS661228	Zaobanyue	Xichuan, Henan	Y&H	50	118.15	39.70	156.02	18.18	1.62	27.12	1.97	10.15	10.18	144.6
AS661229	Baicantiao	Xixia, Henan	Y&H	170	111.85	33.05	161.09	15.3	1.45	26.78	1.38	9.26	10.76	136.34
AS661230	Zhuaziya	Neixiang, Henan	Y&H	170	111.85	33.05	164.58	15.8	1.54	25.82	1.38	10.72	11.47	155.07
AS661231	Baiheshangtou	Neixiang, Henan	Y&H	170	111.85	33.05	158.87	18.29	1.3	25.89	1.09	11.51	10.21	146.12
AS661232	Hongquanmang	Neixiang, Henan	Y&H	170	111.85	33.05	165.53	20.29	1.77	21.58	1.09	10.69	11.28	153.08
AS661233	Yerenmao	Neixiang, Henan	Y&H	77	114.07	32.12	161.72	21.99	1.64	26.62	1.67	11.08	9.67	147.05
AS661234	Xiaozihong	Xinyang, Henan	Y&H	52	115.68	32.18	165.85	20.45	1.52	21.13	1.09	10.37	14.57	141.2
AS661236	Hongmangcao	Gushi, Henan	YTS	52	115.68	32.18	162.68	16.63	1.26	28.97	1.09	11.13	12.12	152.07
AS661237	Huomai	Gushi, Henan	YTS	147	113.32	32.72	162.99	19.16	1.49	26.1	1.09	11.03	12.76	156.4
AS661239	Baihuomai	Miyang, Henan	Y&H	861	110.17	37.77	155.7	18.41	1.6	26.84	1.97	10.03	10.63	143.54
AS661241	Changmangbaike	Mizhi, Shanxi	NW	que	115.22	37.92	159.82	22.66	1.77	21.97	2.4	11.99	10.25	128.78
AS661243	Shihuiyaohuomai	Yanchang, Shanxi	NW	1068	109.32	36.88	175.67	21.35	1.39	25.08	1.09	10.31	14.8	142.81
AS661244	Baitumai	Ansai, Shanxi	NW	1284	108.17	36.93	169.97	21.41	1.3	25.54	1.09	10.66	15.02	144.24
AS661246	Laomai	Wuqi, Shanxi	NW	571	110.57	34.55	162.36	18.46	1.47	19.56	1.38	8.98	11.99	139.97
AS661249	Baimazhamai	Tongguan, Shanxi	NW	500	109.58	34.95	162.36	17.62	1.36	21.69	1.09	8.7	11.83	143.66
AS661250	Siqiangxiaomai	Pucheng, Shanxi	NW	450	109.17	34.75	163.63	17.33	1.49	22.91	1.24	7.98	11.44	141.61
AS661251	Nvdonghong	Fuping, Shanxi	NW	570	110.30	33.67	162.04	18.55	1.23	23.93	1.24	9.15	14.25	144.15
AS661252	Dapushanba	Danfeng, Shanxi	Y&H	705	109.95	33.92	162.68	14.76	1.64	21.71	1.24	9.82	11.89	151.81
AS661253	Liulengmai	Luonan, Shanxi	Y&H	705	109.95	33.92	175.67	22.6	1.49	21.54	1.09	11.46	12.86	147.87
AS661254	Daqimai	Shangxian, Shanxi	Y&H	960	106.52	33.92	162.68	17.17	1.6	21.69	1.24	8.27	10.99	146.17
AS661256	Gouweibabaimai	Fengxian, Shanxi	Y&H	70	114.58	38.15	174.72	24.72	1.49	22.65	1.09	12.51	15.22	137.68
AS661257	Lengshanmai	Xunyang, Shanxi	SWAS	537	106.93	33.00	166.16	21.81	1.58	15.41	1.09	11.49	11.51	150.53
AS661258	Yudongmai	Nanzheng, Shanxi	Y&H	500	109.58	34.95	163.94	16.72	1.54	19.54	1.24	11.1	11.99	150.35
AS661259	Laobaitiaozi	Pucheng, Shanxi	NW	que	108.83	34.25	157.6	19.14	1.26	25.04	1.67	10.17	12.57	146.35
AS661260	Yangmai	Xian, Shanxi	NW	773	110.08	36.87	159.82	18.7	1.62	23.06	1.09	10.04	13.38	137.11
AS661262	Huangwumanglaomai	Yanchuan, Shanxi	NW	830	109.27	35.58	162.99	19.21	1.38	22.95	1.09	10.33	11.99	144.02
AS661263	Sichuanbai	Huangling, Shanxi	NW	830	109.27	35.58	166.8	17.77	1.24	21.41	1.09	7.93	15.41	131.53
AS661264	Siyuehuang	Huangling, Shanxi	NW	1198	107.80	35.22	167.43	20	1.26	26.47	1.09	9.18	13.09	140.78
AS661265	Wuzimai	Changwu, Shanxi	NW	423	108.78	34.60	162.36	18.22	1.21	17.13	1.09	8.02	11.12	141.65
AS661266	Donghuangmai	Sanyuan, Shanxi	NW	358	109.77	34.52	163.63	16.68	1.1	21.63	1.09	7.91	13.57	135.22
AS661267	Baitiaomai	Huaxian, Shanxi	NW	44	115.03	38.03	159.82	15.99	1.17	21.65	1.09	8.46	13.28	143.24
AS661268	Honglanmai	Baishui, Shanxi	NW	690	109.92	35.18	162.36	16.83	1.32	23.41	1.38	8.36	12.22	148.15
AS661270	Laotiaozimai	Chengcheng, Shanxi	NW	131	126.10	47.60	162.99	17.3	1.23	19.89	1.09	8.08	10.96	134.98
AS661274	Yangbaimai	Baiquan, Heilongjiang	NES	225	125.22	49.17	169.65	24.47	1.8	17.8	2.26	12.76	11.76	131.92
AS661275	Guangtou	Nenjiang, Heilongjiang	NES	280	130.28	45.30	166.8	23.92	1.52	33.71	2.26	13.57	12.67	157.73
AS661277	Changsuibaimai	Linkou, Heilongjiang	NES	154	124.83	47.18	166.8	21.74	1.49	28.41	1.97	13.24	14.25	146.5
AS661278	Yangmai	Lalin, Heilongjiang	NES	66	131.98	47.25	174.4	23.19	1.49	27.56	1.97	14	14.48	151.41
AS661279	Youmangbaifu	Fujin, Heilongjiang	NES	120	128.83	45.83	168.06	24.88	1.79	26.21	2.26	14.38	11.86	147.45
AS661280	Fangzhengbendimai	Fangzheng, Heilongjiang	NES	164	126.60	46.00	158.55	14.8	1.71	13.91	1.82	11.67	11.46	107.33
AS661281	Xiaohongmang	Hulan, Heilongjiang	NES	360	128.92	43.12	161.72	18.49	1.43	18.58	1.09	9.76	11.8	135.74
AS661282	Zaoyangmai	Antu, Jilin	NES	35	114.67	37.48	156.97	25.12	1.6	28.65	2.26	13.08	11.8	137.76
AS661283	Daqingmang	Helong, Jilin	NES	218	126.47	43.95	170.28	18.37	1.26	23.34	1.09	10.26	13.09	137.39
AS661284	Xuemai	Jilin	NES	-	121.23	38.82	164.26	21.14	1.3	27.52	1.09	9.39	10.21	148.23
AS661286	Dabaili	Lvda, Liaoning	NW	36	121.95	39.40	160.77	23.83	1.75	26.02	2.4	12.63	13.41	139.41
AS661287	Xinjinyoumang	Xinjin, Liaoning	NW	-	121.70	39.08	166.48	19.71	1.3	23.17	1.09	10.04	14.02	144.58
AS661289	Xiaoyehongmai	Liaodong	NW	-	111.42	41.12	164.58	19.37	1.41	21.41	1.09	9.44	13.8	137.03
AS661290	Shishoumai	Wuchuan, Neimenggu	NS	-	113.15	41.03	164.26	21.25	1.51	17.69	2.4	11.67	15.28	135.36
AS661293	Dahongpao	Jining, Neimenggu	NS	-	113.15	41.03	163.94	20.16	1.49	16.78	2.26	11.71	11.91	138.67
AS661295	Mangmai	Jining, Neimenggu	NS	-	113.17	40.45	162.04	18.55	1.49	18.41	2.26	12.17	11.21	142.81
AS661297	Xiaoriqixiaomai	Fengzhen, Neimenggugu	NS	-	113.17	40.45	158.55	20.87	1.38	21.41	2.84	11.95	9.63	132.77
AS661298	Dahongpao	Fengzhen, Neimenggugu	NS	65	116.22	40.22	166.16	21.53	1.67	19.43	2.26	11.47	11.41	140.79
AS661302	Xiaohongmai	Saxian, Neimenggu	NS	-	113.87	40.87	172.19	20.77	1.77	17.15	2.26	12.29	11.73	143.04
AS661303	Baimangmai	Saxian, Neimenggu	NS	-	111.12	40.75	149.68	18.58	1.38	24.28	2.84	10.27	11.7	125.1
AS661305	Dahongmai	Huhehaoteshi, Neimenggugu	NS	-	106.95	40.33	166.48	20.78	1.51	22.17	1.67	11.98	14.06	144.91
AS661306	Xiaobaisui	Dengkou, Neimenggugu	NS	1877	104.47	34.85	164.89	21.98	1.56	24.34	2.26	12.85	12.54	140.4
AS661310	Lvjiankou	Zhangxian, Gansu	NWS	2315	104.02	34.43	162.99	18.68	1.47	20.45	2.26	11.92	11.25	141.46
AS661311	Laohongmai	Minxian, Gansu	NWS	-	116.70	39.50	158.24	19.92	1.71	20.39	2.69	11	8.89	118.37
AS661315	Xiaohemai	Yongdeng, Gansu	NWS	1690	103.93	36.37	163.94	22.83	1.66	20.15	2.26	12.95	12.89	140.06
AS661316	Qingmangmai	Gaolan, Gansu	NWS	1690	103.93	36.37	166.8	23.66	1.62	20.63	2.26	12.26	11.38	141.89
AS661317	Xiaoheshangtou	Gaolan, Gansu	NWS	1690	103.93	36.37	166.48	22.53	1.77	20.28	2.26	11.26	11.47	131.38
AS661318	Qitoumai	Gaolan, Gansu	NWS	1690	103.93	36.37	168.7	22.29	1.62	16.11	2.26	10.74	12.96	127.87
AS661319	Waibozi	Gaolan, Gansu	NWS	2136	103.50	35.53	167.75	23.87	1.62	17.93	2.26	12.76	12.83	136.44
AS661320	Xiaomai	Hezheng, Gansu	NWS	2468	103.40	35.67	166.8	22.17	1.9	15.17	2.26	13.88	11.86	134.5
AS661321	Baijiankou	Dongxiangzuzizhixian, Gansu	NWS	1364	103.08	38.62	166.16	22.35	1.84	26.69	1.53	11.13	12.31	138.03
AS661324	Bailaolaibian	Yongchang, Gansu	NWS	96	114.55	38.45	165.21	18.23	1.88	18.34	2.26	11.37	12.54	145.02
AS661327	Baidatou	Yumen, Gansu	NWS	2312	-	-	-	-	-	-	-	-	-	-
AS661329	Shanxihong	Yumen, Gansu	NWS	1178	95.77	40.53	164.26	19.91	1.56	20.78	2.26	11.24	13.68	125.19
AS661331	Xiaobaimai	Anxi, Gansu	NWS	1100	107.90	35.98	158.87	21.11	1.67	27.58	2.84	11.13	10.18	136.09
AS661332	Shanxibaimai	Qingyang, Gansu	NW	1100	107.90	35.98	175.04	17.71	1.11	22.91	1.09	9.23	15.93	128
AS661333	Baimangmai	Qingyang, Gansu	NW	922	108.00	35.42	166.8	19.12	1.13	20.32	1.09	7.86	15.64	127.68
AS661334	Hongmazha	Ningxian, Gansu	NW	1453	108.37	35.48	173.45	18.46	1.41	24.6	1.09	8.52	13.12	134.11
AS661335	Baiqimai	Zhengning, Gansu	NW	1240	107.30	36.57	162.36	18.96	1.17	17.63	1.09	7.36	12.73	119.35
AS661337	Xiaoqingmang	Huanxian, Gansu	NW	1297	108.02	35.82	168.06	17.65	1.15	20.58	1.09	7.76	13.96	123.61
AS661338	Hongqimai	Heshui, Gansu	NW	1200	105.67	34.87	166.48	19.86	1.21	22.26	1.09	8.1	15.54	132.95
AS661341	Laomangmai	Qinan, Gansu	SWAS	75	114.68	38.35	168.7	19.13	1.21	19.19	1.09	9.2	15.57	140.14
AS661350	Dabaimai	Hualong, Qinghai	Q&T	-	86.13	41.77	165.21	25.76	1.94	18.47	2.26	12.99	11.89	126.77
AS661357	Kuchebaidongmai	Kuche, Xinjiang	XJ	1013	82.62	41.53	172.5	21.14	1.32	20.42	1.09	11.31	13.56	132.77
AS661358	Reyimuxia	Xinhe, Xinjiang	XJ	981	82.78	41.23	-	-	-	-	-	-	-	-
AS661359	Mailuowaxi	Shaya, Xinjiang	XJ	1360	77.32	37.10	175.99	21.42	1.39	23.8	1.09	9.74	11.47	139.22
AS661366	Haaretuosiman	Yecheng, Xinjiang	XJ	1360	77.32	37.10	-	-	-	-	-	-	-	-
AS661367	Heidongmai	Yecheng, Xinjiang	XJ	4641	86.22	44.30	-	-	-	-	-	-	-	-
AS661373	Jiuhaomai	Wujiang, Jiangsu	YTS	70	114.58	38.15	156.65	22.52	1.79	27.17	2.26	10.93	12.47	143.79
AS661374	Haiyanzhong	Wujiang, Jiangsu	YTS	4	120.62	31.30	161.41	23.56	1.54	27.36	1.67	12.74	11.18	152.36
AS661375	Cuodaosiguatou	Wuxian, Jiangsu	YTS	4	120.62	31.30	160.77	19.22	1.67	21.06	1.82	9.44	12.15	147.25
AS661376	Youmangzipi	Wuxian, Jiangsu	YTS	4	121.08	31.45	163.31	22.99	1.64	32.3	1.82	14.05	11.38	167.82
AS661378	Liuzhutou	Taicang, Jiangsu	YTS	4	121.08	31.45	160.14	19.9	1.62	27.6	1.67	8.9	9.86	146.18
AS661379	Yangxiaomai	Taicang, Jiangsu	YTS	5	120.73	31.67	160.46	18.31	1.36	26.54	1.38	11.58	10.44	150.34
AS661380	Jianzituan	Changshu, Jiangsu	YTS	5	120.73	31.67	157.6	19.06	1.52	22.82	1.67	10.55	9.54	143.89
AS661381	Heshangtou	Changshu, Jiangsu	YTS	5	120.27	31.55	160.46	19.92	1.67	22.6	2.11	9.35	11.05	143.73
AS661382	Baisiguatou	Wuxi, Jiangsu	YTS	5	120.27	31.55	162.04	19.05	1.47	25.75	1.82	12.27	13.06	153.24
AS661383	Baichangshao	Wuxi, Jiangsu	YTS	5	120.27	31.55	158.87	21.09	1.54	32.12	1.97	12.82	12.31	168.82
AS661384	Jianfangzhu	Wuxi, Jiangsu	YTS	44	115.03	38.03	157.6	18.3	1.62	27.08	1.24	11.05	10.6	149.73
AS661385	Dongmai	Wuxi, Jiangsu	YTS	5	120.27	31.55	163.31	18.91	1.52	25.89	1.09	12.78	10.99	161.01
AS661386	Huangkefangliuzhu	Wuxi, Jiangsu	YTS	5	120.27	31.55	161.09	20.71	1.71	24.36	2.11	9.67	10.44	153.33
AS661387	Jiangyinbaimai	Wuxi, Jiangsu	YTS	5	120.27	31.55	161.09	23.43	1.64	29.43	1.67	12.66	12.09	156.9
AS661388	Honghuaqimai	Wuxi, Jiangsu	YTS	5	120.27	31.55	161.72	19.8	1.62	22.78	1.82	10.26	11.15	142.73
AS661389	Huangfangzhu	Wuxi, Jiangsu	YTS	7	120.28	31.67	159.51	19	1.66	27.69	1.38	10.42	10.54	144.97
AS661390	Baikeliuzhutou	Jiangyin, Jiangsu	YTS	7	120.28	31.67	159.51	19.27	1.73	26.62	1.97	8.99	11.05	149.34
AS661392	Yejihong	Jiangyin, Jiangsu	YTS	6	119.80	31.37	159.19	20.33	1.51	27.95	1.67	13.11	10.83	153.44
AS661393	Zaoxiaomai	Yixing, Jiangsu	YTS	6	119.80	31.37	163.63	21.18	1.45	25.58	1.82	13.52	12.15	151.1
AS661394	Annanmai	Yixing, Jiangsu	YTS	6	119.80	31.37	157.6	21.7	1.71	26.23	2.11	10.53	10.89	137.01
AS661395	Baiguanglangtou	Yixing, Jiangsu	YTS	44	115.03	38.03	160.46	19.31	1.51	25.19	2.11	11.83	14.19	151.94
AS661396	Huoshaotian	Yixing, Jiangsu	YTS	8	118.88	31.32	160.14	18.46	1.34	21.02	1.53	11.69	10.57	131.49
AS661397	Hongmangzaoxiaomai	Gaochun, Jiangsu	YTS	5	119.48	31.43	155.38	17.61	1.52	20.32	1.97	11.28	10.18	140.98

Supplemental Table S1 Information of the 707 wheat accessions assessed in the present study.

Accession No.	Landrace name	Origin	Geographic distribution[†]	Atitude (m)	East longitude (°)	Northern Latitude (°)	BLUP of 8 agronomic traits[‡]							
							FD	FLL	FLW	PDL	SH	SL	TN	PH
AS661398	Guangdezao	Liyang, Jiangsu	YTS	8	119.58	31.98	156.02	17.89	1.43	28.36	1.24	11.8	11.02	149.4
AS661400	Luogengqing	Danyang, Jiangsu	YTS	8	119.58	31.98	163.63	20.6	1.39	28.78	1.53	11.37	11.57	154.76
AS661401	Balangtou	Danyang, Jiangsu	YTS	8	119.58	31.98	157.6	18.98	1.51	30.43	1.82	10.68	12.28	153.27
AS661402	Zaobaimangmai	Danyang, Jiangsu	YTS	4	119.40	32.20	158.55	18.3	1.49	25.1	1.82	12.66	11.21	156.4
AS661404	Chixiaomai	Zhenjiang, Jiangsu	YTS	4	119.82	32.27	160.46	22.73	1.54	34.51	2.11	13.48	10.67	157.1
AS661405	Yanglazi	Yangzhong, Jiangsu	YTS	4	119.82	32.27	163.63	18.91	1.51	24.32	2.11	10.28	10.21	145.74
AS661406	Hongxiaomai	Yangzhong, Jiangsu	YTS	8	118.63	32.05	158.55	19.88	1.45	26.71	1.53	12.04	10.76	155.21
AS661408	Ziganzi	Jiangpu, Jiangsu	YTS	50	118.15	39.70	161.72	21.52	1.67	25.19	1.67	8.91	11.47	147.11
AS661409	Xiaohongtou	Liuhe, Jiangsu	YTS	2	121.65	31.80	161.41	18.03	1.39	28.56	1.38	10.9	9.47	145.63
AS661411	Changqibaike	Qidong, Jiangsu	YTS	4	121.15	31.83	159.51	18.16	1.32	29.01	1.67	11.2	11.31	151.31
AS661412	Luopangtou	Haimen, Jiangsu	YTS	2	120.85	32.02	159.82	19.1	1.58	26.71	1.53	9.19	12.44	140.44
AS661413	Youmangsilengtou	Nantong, Jiangsu	YTS	2	120.85	32.02	160.14	19.75	1.3	28.51	1.67	11.62	11.8	157.02
AS661414	Daoshuibai	Nantong, Jiangsu	YTS	2	120.85	32.02	157.92	18.6	1.47	27.02	1.97	11.78	9.96	154.09
AS661415	Changsuibaike	Nantong, Jiangsu	YTS	2	120.85	32.02	159.19	20.09	1.79	25.17	2.26	8.84	11.02	141.5
AS661416	Baichaoyu	Nantong, Jiangsu	YTS	2	120.85	32.02	157.92	17.68	1.39	26.23	1.53	10.4	10.54	138.58
AS661417	Hongkechangmang	Nantong, Jiangsu	YTS	2	120.85	32.02	161.41	20.47	1.43	25.15	1.38	12.35	11.83	150.64
AS661418	Baijiaozi	Nantong, Jiangsu	YTS	4	121.18	32.18	165.21	21.28	1.49	26.65	1.67	11.46	15.19	154.45
AS661419	Zaowutian	Rudong, Jiangsu	YTS	35	115.52	38.85	157.92	18.71	1.66	25.91	2.26	9.27	11.15	143.08
AS661420	Caizihuang	Jingjiang, Jiangsu	YTS	5	119.17	32.28	162.04	21.67	1.84	20.82	1.97	9.96	11.6	146.37
AS661421	Qingmangzi	Yizheng, Jiangsu	YTS	6	119.43	32.40	157.29	18.78	1.51	24.65	1.97	13.49	12.22	160.41
AS661423	Huaqixiaomai	Hanjiang, Jiangsu	YTS	6	119.43	32.40	153.48	21.73	2.01	27.8	1.67	13.32	9.44	149.56
AS661425	Mangkuazi	Hanjiang, Jiangsu	YTS	4	119.87	32.97	159.82	18.79	1.62	23.95	1.97	8.96	10.63	143.8
AS661426	Dahuangpi	Xinghua, Jiangsu	YTS	5	119.42	32.78	159.51	17.5	1.45	29.58	1.82	10.58	10.28	152.3
AS661427	Wumangmai	Gaoyou, Jiangsu	YTS	5	119.42	32.78	159.51	19.09	1.34	28.97	1.82	11.47	11.15	152.51
AS661428	Baimangzi	Gaoyou, Jiangsu	YTS	6	119.30	33.23	155.07	19.54	1.86	26.17	1.53	12.36	10.96	147.46
AS661430	Dahongpi	Baoying, Jiangsu	YTS	5	120.45	33.20	163.31	17.96	1.26	25.25	1.09	8.41	11.54	147.05
AS661431	Meixiuhuang	Dafeng, Jiangsu	YTS	2	119.80	33.47	155.38	20.24	1.95	27.47	1.67	13.03	10.7	155.62
AS661434	Yuhuazi	Jianhu, Jiangsu	YTS	35	115.52	38.85	163.31	17.41	1.43	26.97	1.24	9.82	11.05	147.11
AS661435	Zuantoubaike	Binhai, Jiangsu	Y&H	33	118.75	34.52	160.46	18.71	1.41	28.93	1.09	10.04	11.93	151.19
AS661436	Hongmai	Donghai, Jiangsu	Y&H	35	116.85	34.73	157.92	16.66	1.1	22.19	1.24	9.5	12.38	148.63
AS661439	Sanyuehuang	Peixian, Jiangsu	Y&H	35	116.85	34.73	162.04	19.04	1.34	26.45	1.09	9.55	13.31	144.08
AS661440	Xiaolihong	Peixian, Jiangsu	Y&H	35	116.85	34.73	163.31	18.23	1.36	27.88	1.09	9.11	13.64	150.52
AS661441	Youzimai	Peixian, Jiangsu	Y&H	58	117.22	34.23	162.36	17.56	1.52	29.78	1.82	8.91	10.52	137.14
AS661442	Tutoumai(Jia)	Tongshan, Jiangsu	Y&H	58	117.22	34.23	167.75	18.33	1.66	23.02	1.09	10.01	14.57	148.78
AS661443	Baiyouzi	Tongshan, Jiangsu	Y&H	58	117.22	34.23	163.31	17.23	1.3	22.93	1.09	7.39	10.54	140.13
AS661444	Baisuibai	Tongshan, Jiangsu	Y&H	21	117.75	33.90	161.72	19.99	1.49	21.8	1.97	10.08	10.31	130.94
AS661445	Baisuihong	Suining, Jiangsu	YTS	22	117.95	34.30	156.97	18.44	1.32	24.65	1.09	9.81	10.15	130.32
AS661446	Hongshimai	Pixian, Jiangsu	Y&H	46	116.28	39.98	161.09	19.39	1.71	30.21	1.82	9.64	11.44	149.95
AS661447	Xiaohongmai	Donghai, Jiangsu	Y&H	15	118.72	33.70	162.36	18.6	1.28	21.28	1.24	9.05	12.67	144.9
AS661448	Xiaoyuhua	Siyang, Jiangsu	YTS	4	119.25	34.38	164.58	18.08	1.3	25.52	1.09	10.25	12.34	147.69
AS661449	Daliuleng	Guanyun, Jiangsu	Y&H	4	119.25	34.38	164.89	18.86	1.43	25.32	1.24	9.11	11.86	144.84
AS661450	Dayangxiaomai	Guanyun, Jiangsu	Y&H	10	119.07	33.67	163.94	23.66	2.14	32.93	2.26	14.33	11.54	154.91
AS661451	Baiheshangtou	Huaiyin, Jiangsu	YTS	7	119.17	33.53	162.36	19.02	1.41	29.71	1.09	12.97	11.99	159.98
AS661452	Shunshuihong	Huaian, Jiangsu	YTS	7	119.27	33.78	153.8	19.32	1.38	25.54	1.53	11.39	12.02	139.31
AS661453	Xiaoyuhua	Lianshui, Jiangsu	YTS	4	121.23	31.00	163.94	19.64	1.3	21.5	1.09	10.52	12.34	146.92
AS661454	Putaomai	Songjiang, Shanghai	YTS	4	121.12	31.15	161.41	18.27	1.51	20.63	1.97	7.59	10.8	138.06
AS661455	Baijiaomai	Qingpu, Shanghai	YTS	5	121.25	31.38	161.09	18.31	1.64	26.25	1.82	9.43	13.23	150.15
AS661458	Zhangsihong	Jiading, Shanghai	YTS	10	116.92	38.33	157.6	18.02	1.66	23.76	1.82	10.47	10.67	152.68
AS661459	Waiguoyazuitou	Chongming, Shanghai	YTS	160	118.58	30.08	156.97	21.14	1.73	26.36	2.11	9.16	9.81	142.01
AS661460	Huxumai	Jixi, Anhui	YTS	193	118.53	30.28	159.82	19.74	1.47	22.21	1.82	9.29	10.31	138.22
AS661461	Wugongmai	Jingde, Anhui	YTS	23	116.68	30.13	160.46	21.39	1.52	23.78	2.26	9.4	10.31	133.35
AS661462	Jiangxizao	Wangjiang, Anhui	YTS	39	116.28	30.43	158.87	20.82	1.41	34.01	1.09	14.63	10.67	171.75
AS661463	Wugongbian	Taihu, Anhui	YTS	14	117.85	30.65	156.02	20.7	1.66	26.45	1.67	12.42	10.68	142.94
AS661465	Heshangtou	Qingyang, Anhui	YTS	10	118.37	31.72	163.31	19.13	1.62	23.1	2.11	10.69	12.38	147.12
AS661469	Daheshangtou	Hexian, Anhui	YTS	26	117.55	33.53	158.55	19.7	1.52	28.32	1.97	9.88	9.47	159.63
AS661470	Hongmai	Lingbi, Anhui	YTS	38	115.35	33.27	156.02	21.44	1.38	30.14	1.53	11.38	13.28	153.3
AS661472	Feixisanyuehuang	Jieshou, Anhui	YTS	7	116.83	38.58	159.51	20.59	1.49	21.47	1.24	10.44	10.05	149.6
AS661473	Dongjiaqiaoxiaomai	Hangzhou, Zhejiang	YTS	9	120.13	30.18	153.17	24.72	1.71	34.56	2.84	14.45	10.44	147.88
AS661474	Shangbaosanyuehuang	Hangzhou, Zhejiang	YTS	9	120.13	30.18	156.97	21.01	1.66	27.32	1.82	11.03	9.57	136.34
AS661475	Shangsasongputou	Hangzhou, Zhejiang	YTS	9	120.13	30.18	162.04	19.25	1.58	26.95	2.26	9.35	10.92	149.33
AS661476	Linpingyangmai	Hangzhou, Zhejiang	YTS	7	120.28	30.42	159.82	21.78	1.51	26.15	1.97	12.23	11.86	156.36
AS661477	Baixu	Chongde, Zhejiang	YTS	7	119.93	30.05	161.72	21.73	1.73	23.95	2.26	8.38	9.99	146.62
AS661478	Hongkemagongzhong	Fuyang, Zhejiang	YTS	7	119.93	30.05	155.38	23.08	1.77	25.71	2.11	11.28	10.76	135.91
AS661479	Baikeguangtou	Fuyang, Zhejiang	YTS	7	119.93	30.05	156.65	19.85	1.6	27.15	2.11	11.35	10.44	155.37
AS661480	Honglizitou	Fuyang, Zhejiang	YTS	7	119.93	30.05	159.19	20.61	1.62	24.91	1.97	10.82	11.12	145.31
AS661481	Xumai	Fuyang, Zhejiang	YTS	7	119.93	30.05	160.46	19.43	1.64	27.19	1.82	8.83	12.04	135.66
AS661482	Huoshaomai	Fuyang, Zhejiang	YTS	7	116.83	38.58	160.77	20.59	1.58	24.3	1.97	8.01	10.34	138.21
AS661483	Hongkewugong	Shouchang, Zhejiang	YTS	-	119.42	30.18	161.72	18.75	1.58	22.89	2.11	10.21	12.78	159.22
AS661484	Guangmai	Yuqian, Zhejiang	YTS	-	119.42	30.18	159.19	17.56	1.84	24.78	1.97	9.37	10.15	135.61
AS661485	Fangpu	Xiaofeng, Zhejiang	YTS	25	119.68	29.82	158.55	20.77	1.99	25.28	1.82	8.81	12.67	144.08
AS661488	Tiedingmai	Tonglu, Zhejiang	YTS	112	119.03	29.62	159.82	19.95	1.56	26.3	1.97	11.6	11.47	151.66
AS661490	Liulengmaomai	Chunan, Zhejiang	YTS	-	120.10	30.87	159.51	18.8	1.75	21.97	2.11	9.37	9.92	138.14
AS661492	Puzhumai	Wuxing, Zhejiang	YTS	3	122.10	30.02	161.72	21.19	1.64	20.87	2.11	6.9	13.22	136.4
AS661493	Baixuxiaomai	Dinghai, Zhejiang	YTS	2	122.22	30.23	164.26	21.38	1.52	24.76	1.97	13.72	10.83	148.53
AS661494	Wumangmai	Daishan, Zhejiang	YTS	5	121.87	29.48	161.72	17.57	1.49	22.41	1.53	12.16	9.31	146.36
AS661495	Maofangtou	Xiangshan, Zhejiang	YTS	5	121.87	29.48	159.19	23.4	1.79	24.93	2.11	10.88	10.76	147.6
AS661496	Huashupu	Xiangshan, Zhejiang	YTS	7	116.83	38.58	161.09	21.22	1.66	25.06	2.11	9.1	11.02	144.54
AS661497	Baitengkousui	Yuyao, Zhejiang	YTS	5	121.15	30.05	159.82	20.11	1.82	24.56	2.4	12.56	9.7	150.15
AS661498	Tiezuantou	Yuyao, Zhejiang	YTS	5	121.15	30.05	159.82	18.29	1.43	22.02	2.11	11.31	11.05	141.02
AS661499	Guangtoumai	Yuyao, Zhejiang	YTS	6	120.58	30.00	162.04	19.23	1.45	23.73	2.26	9.36	12.83	150.2
AS661500	Laolaibai	Shaoxing, Zhejiang	YTS	6	120.58	30.00	166.8	22.28	1.66	25.12	2.11	11.15	10.28	144.3
AS661501	Yangxiaomai	Shaoxing, Zhejiang	YTS	500	111.20	34.80	163.94	21.39	1.6	25.56	2.26	11.23	12.76	156.61
AS661502	Xianjumai	Sanmen, Zhejiang	YTS	5	121.33	28.22	159.51	19.53	1.62	28.41	1.38	11.9	11.18	156.13
AS661503	Baimai	Wenling, Zhejiang	YTS	51	121.03	29.13	152.53	20.95	1.77	29.23	2.11	13.05	11.57	151.87
AS661504	Songhuapu	Tiantai, Zhejiang	YTS	51	121.03	29.13	158.24	18.3	1.54	24.19	1.97	6.82	11.18	133.5
AS661505	Zaomaiguang	Tiantai, Zhejiang	YTS	60	119.92	28.45	158.24	17.06	1.43	20.26	1.67	9.29	13.17	140.42
AS661506	Tiantaimai	Lishui, Zhejiang	YTS	9	117.10	38.05	162.36	19.71	1.6	22.78	1.97	8.18	11.7	148
AS661507	Qingtianmai	Lishui, Zhejiang	YTS	127	119.48	28.50	158.87	20.52	2.25	18.04	2.26	10.65	8.34	135.16
AS661509	Baihuamai	Songyang, Zhejiang	YTS	151	120.07	28.65	160.46	17.82	1.71	23.65	2.11	11.73	9.83	157.69
AS661510	Hongtoumai	Jinyun, Zhejiang	YTS	45	119.65	29.12	156.65	17.5	1.54	24.43	1.97	11.44	9.79	148.72
AS661511	Xiyangmai	Jinhua, Zhejiang	YTS	45	119.65	29.12	160.46	23.03	1.84	22.91	2.11	9.31	11.7	144.03
AS661512	Dongyanghongpu	Jinhua, Zhejiang	YTS	45	119.65	29.12	160.14	22.1	1.54	23.6	1.97	6.96	9.5	134.2
AS661513	Hongmai	Jinhua, Zhejiang	YTS	45	119.65	29.12	158.87	20.21	1.45	26.8	2.11	12.08	12.54	149.59
AS661514	Yejimai	Jinhua, Zhejiang	YTS	70	119.88	29.45	158.55	19.36	1.64	23.69	2.26	8.74	9.54	138.94
AS661515	Songpuhongtou	Pujiang, Zhejiang	YTS	94	120.22	29.27	156.65	19.58	1.64	21.21	2.11	8.3	10.8	142.88
AS661516	Baipuzaoxiaomai	Dongyang, Zhejiang	YTS	95	118.62	28.73	161.41	20.99	1.82	23.04	2.11	8.46	13.51	142.19
AS661518	Jiyumai	Jiangshan, Zhejiang	YTS	-	116.83	38.33	162.99	21.98	1.58	22.67	2.26	8.19	13.6	152.92
AS661520	Chanmai	Changshan, Zhejiang	YTS	95	118.52	28.88	159.19	19.46	1.66	20.73	1.97	8.03	10.38	141.51
AS661521	Wugongmai	Changshan, Zhejiang	YTS	7	119.93	30.05	160.14	20.31	1.64	24.78	1.97	10.98	11.28	150.86
AS661523	Dingmanghongke	Fuyang, Zhejiang	YTS	112	119.03	29.62	159.51	20.86	1.64	26.32	1.97	9.94	11.25	150.57
AS661524	Heshangmai	Chunan, Zhejiang	YTS	4	120.53	30.63	160.14	21.4	1.8	23.65	1.53	8.63	9.7	144.59
AS661525	Baipusui	Tongxiang, Zhejiang	YTS	20	119.68	30.63	160.46	19.97	1.71	23.41	2.11	8.29	10.73	140.86
AS661526	Yangmai	Anji, Zhejiang	YTS	3	120.68	30.53	153.29	22.9	1.78	28.87	2.51	12.21	8.95	124.2
AS661527	Tiegengqing	Haining, Zhejiang	YTS	6	120.58	30.00	163.31	18.7	1.39	20.19	2.26	7.78	12.57	141.35
AS661528	Zaoxiaomai	Shaoxing, Zhejiang	YTS	51	121.03	29.13	156.02	18.09	1.3	22.89	1.97	10.28	12.15	142.12
AS661529	Shelidong	Tiantai, Zhejiang	YTS	17	120.28	28.15	156.34	17.36	1.45	24.71	1.97	8.59	9.83	128.1
AS661530	Baimai	Qingtian, Zhejiang	YTS	24	115.75	37.50	160.14	23.83	1.71	21.39	2.11	9.96	9.83	142.78
AS661531	Linhaichangbaican	Jinyun, Zhejiang	YTS	45	119.65	29.12	158.24	19.43	1.66	22.86	2.11	10.81	10.05	138.18
AS661532	Baipu(Luoqing)	Jinhua, Zhejiang	YTS	65	120.07	29.30	160.14	19.71	1.58	19.65	1.97	9.02	11.31	141.23
AS661533	Baikezao	Yiwu, Zhejiang	YTS	2	121.02	30.70	162.68	18.06	1.75	22.65	2.26	11.13	11.83	141.29
AS661534	Canshumai	Pinghu, Zhejiang	YTS	32	116.27	28.37	154.43	20.01	1.9	16.58	2.11	12.67	10.76	114.6
AS661535	Paozimai	Jinxian, Jiangxi	YTS	71	117.95	28.43	152.53	18.22	1.6	22.56	2.4	12.77	8.37	128.49
AS661536	Niqiumai	Shangrao, Jiangxi	YTS	-	113.38	31.72	160.77	19.54	1.6	25.99	1.82	13.18	10.6	150.76
AS661541	Wangshuibai	Suixian, Hubei	YTS	110	111.87	26.58	160.77	19	1.71	23.73	1.97	9.05	11.28	143.01
AS661542	Qiyanghongke	Qiyang, Hunan	YTS	153	109.80	27.87	159.51	20.08	1.64	22.93	1.24	11.26	9.86	140.91
AS661543	Mayangyangmai	Mayang, Hunan	YTS	110	111.87	26.58	155.07	18.39	1.41	26.56	1.67	11.69	12.83	132.87
AS661544	Hongkemai	Qiyang, Hunan	YTS	23	115.58	37.57	162.99	17.52	1.47	26.6	1.67	11.91	11.15	149.41
AS661545	Youmanghongke	Rucheng, Hunan	SAS	268	109.85	29.02	159.19	18.74	1.45	24.62	1.97	14.13	10.89	157.22
AS661547	Heshangmai	Yongshun, Hunan	YTS	360	116.75	25.68	159.19	20.86	1.71	23.82	1.38	10.4	10.76	148.17
AS661549	Baikeyoumang	Liancheng, Fujian	SAS	65	118.70	25.45	150	21.03	1.88	28.8	2.26	13.62	10.41	140.73
AS661550	Hongkexiaomai	Xianyou, Fujian	SAS	45	118.17	25.07	149.68	24.65	2.12	27.17	2.69	11.98	9.57	131.67
AS661552	Hongkemai	Anxi, Fujian	SAS	5	115.63	22.95	147.78	24.32	1.92	29.21	2.84	11.35	9.34	131.1
AS661554	Youmai	Lufeng, Guangdong	SAS	2	113.37	22.52	149.04	25.32	1.75	31.95	2.55	13.24	10.73	140.82
AS661555	Datianquxiaomai	Zhongshan, Guangdong	SAS	22	111.77	23.15	150.95	23.44	1.79	25.15	2.11	11.63	10.38	130.31
AS661557	Mianmai	Deqing, Guangdong	SAS	14	113.02	23.70	159.19	21	1.56	23.17	2.26	12.03	10.26	151.89
AS661558	Qingyuanxiaomai	Qingyuan, Guangdong	SAS	30	113.40	24.17	150.63	24.95	1.64	30.54	2.69	13.4	10.47	136.21
AS661560	Huoshaomai	Yingde, Guangdong	SAS	26	115.88	37.33	156.34	25.3	1.77	29.78	2.11	13.96	12.54	148.19
AS661561	Kangmai	Tancheng, Guangdong	SAS	5	115.63	22.95	152.53	23.84	1.95	27.99	2.4	11.44	10.34	135.24

Supplemental Table S1 Information of the 707 wheat accessions assessed in the present study.

Accession No.	Landrace name	Origin	Geographic distribution†	Atitude (m)	East longitude (°)	Northern Latitude (°)	BLUP of 8 agronomic traits‡							
							FD	FLL	FLW	PDL	SH	SL	TN	PH
AS661562	Baikexiaomai	Lufeng, Guangdong	SAS	80	116.68	24.35	148.73	23.98	1.94	25.8	2.69	11.19	11.31	129.79
AS661563	Zaoxiaomai	Dapu, Guangdong	SAS	10	116.17	23.30	152.85	19.69	1.82	27.17	2.84	10.73	10.44	135.54
AS661564	Puningxiaomai	Puning, Guangdong	SAS	10	116.17	23.30	160.46	20.37	1.71	25.47	1.24	12.15	11.15	153.05
AS661565	Wuxuxiaomai	Puning, Guangdong	SAS	49	109.62	23.12	152.85	22.85	1.69	27.8	2.11	12.65	13.18	146.06
AS661566	Zhangmuxiaomai	Guixian, Guangxi	SAS	4352	110.08	23.40	151.9	23.13	1.73	27.62	2.84	10.92	10.54	137.64
AS661567	Guipingxiaomai	Guiping, Guangxi	SAS	103	110.63	24.63	155.38	22.43	1.88	29.1	2.26	12.43	11.31	144.14
AS661569	Guangtoumai	Pingle, Guangxi	SAS	373	106.77	31.85	160.77	20.02	1.56	25.02	1.53	10.15	10.76	142.19
AS661571	Sanyuehuang	Bazhong, Sichuan	SWAS	453	107.85	31.08	157.6	21.83	1.66	25.34	1.97	10.3	11.83	134.66
AS661573	Baimaizi	Kaijiang, Sichuan	SWAS	24	116.65	39.90	160.77	23.48	1.8	29.6	2.26	11.33	10.73	153.34
AS661574	Honghuamaizhong	Wanyuan, Sichuan	SWAS	255	106.97	30.25	160.46	21.38	1.79	24.47	2.26	9.14	11.09	142.36
AS661575	Guangtoumai	Quxian, Sichuan	SWAS	287	104.62	28.77	159.51	23.25	1.86	22.45	2.11	10.13	11.73	143.39
AS661580	Dongxumai	Yibin, Sichuan	SWAS	287	104.62	28.77	161.09	23	2.12	27.23	2.26	9.8	12.05	152.04
AS661581	Zhudongmai	Yibin, Sichuan	SWAS	287	104.62	28.77	160.46	23.42	1.86	25.08	1.67	9.57	11.18	145.11
AS661582	Wuyangmai	Yibin, Sichuan	SWAS	276	104.92	28.58	155.7	27.05	1.88	26.71	1.97	11.44	10.99	131.49
AS661583	Huangmaizi	Changning, Sichuan	SWAS	245	105.45	28.90	156.65	21.15	1.92	22.3	2.26	10.21	10.44	139.76
AS661584	Yuzuiweimai	Luxian, Sichuan	SWAS	245	105.45	28.90	161.41	18.78	1.71	21.17	1.82	8.46	12.31	139.72
AS661585	Dahuangmai	Luxian, Sichuan	SWAS	245	105.45	28.90	160.46	22.61	1.67	26.39	2.26	9.63	13.3	140.32
AS661586	Youtiaomai	Luxian, Sichuan	SWAS	259	105.07	28.73	160.14	23.48	1.69	28.82	2.26	10.16	12.64	143.38
AS661587	Dahonghua	Jiangan, Sichuan	SWAS	27	114.37	37.35	156.34	21.29	1.66	21.06	2.26	10.94	11.47	134.61
AS661588	Nanximai	Gaoxian, Sichuan	SWAS	338	104.52	28.43	158.24	22.13	1.6	26.65	1.53	10.95	12.18	140.79
AS661589	Guangtoumai	Gaoxian, Sichuan	SWAS	338	104.52	28.43	156.97	21.67	1.52	23.86	1.97	9.77	12.52	134.5
AS661590	Dongmaidong	Qingfu, Sichuan	SWAS	425	104.50	28.17	162.04	23.09	1.58	24.58	2.26	7.77	12.86	135.31
AS661592	Zaohonghua	Yunlian, Sichuan	SWAS	237	105.37	28.77	156.65	24.85	1.6	21.6	1.97	10.74	11.12	141.63
AS661593	Zaohuangmai	Naxi, Sichuan	SWAS	226	105.83	28.80	154.12	22.25	1.64	26.32	2.55	10.18	11.02	141.46
AS661594	Yizaixiaomai	Hejiang, Sichuan	SWAS	431	105.02	30.27	157.6	20.22	1.67	26.73	2.11	10.11	10.57	142.71
AS661596	Hongtiaomai	Leyu, Sichuan	SWAS	322	104.65	29.52	158.55	22.2	1.54	23.91	1.97	10.36	12.44	138.64
AS661597	Guangtou	Weiyuan, Sichuan	SWAS	414	103.83	30.05	152.21	21.67	1.54	25.71	2.11	9.81	10.63	126.71
AS661598	Guangtoumai	Meishan, Sichuan	SWAS	1384	102.18	27.42	155.38	23.21	1.75	24.6	2.26	10.83	12.31	129.81
AS661599	Yupi	Dechang, Sichuan	SWAS	27	114.37	37.35	148.41	24.73	2.01	17.82	2.26	10.83	8.12	103.89
AS661600	Baimai	Huili, Sichuan	SWAS	233	106.63	30.47	156.65	20.82	1.82	29.23	2.11	8.73	12.12	140.03
AS661602	Hongxumai	Guangan, Sichuan	SWAS	575	106.40	31.52	161.41	18.27	1.45	18.43	1.82	9.22	10.12	130.4
AS661604	Doumai	Yilong, Sichuan	SWAS	530	104.73	31.78	155.38	19.95	1.67	23.99	2.11	9.65	11.67	146.42
AS661605	Lushanmai	Jiangyou, Sichuan	SWAS	475	105.15	31.65	161.09	21.95	1.66	27.65	2.11	10.9	10.86	142.69
AS661606	Lanmai	Zitong, Sichuan	SWAS	478	105.58	30.50	164.58	22.72	1.73	22	2.11	11.23	13.47	143.22
AS661609	Tuotuomai	Suining, Sichuan	SWAS	395	105.70	30.77	156.65	25.37	1.73	19.73	1.97	10.48	11.38	138.45
AS661610	Honghuaguangtou	Pengxi, Sichuan	SWAS	330	105.55	30.85	159.19	23.17	1.88	26.19	2.11	9.34	11.44	136.49
AS661611	Wulangxiexiaomai	Shehong, Sichuan	SWAS	430	104.67	31.03	156.34	22.95	1.8	24.86	2.26	10.53	10.28	136.99
AS661612	Changxuxuqiaomai	Zhongjiang, Sichuan	SWAS	378	105.08	31.08	156.97	21.92	1.67	21.76	1.82	11.97	11.21	135.1
AS661613	Qingquanxiaomai	Santai, Sichuan	SWAS	32	114.93	37.60	148.73	26.02	1.99	27.71	2.84	12.83	10.05	125.84
AS661615	Yuweimai	Dazu, Sichuan	SWAS	218	106.25	30.00	160.14	24.27	1.54	22.19	2.26	11.01	13.09	145.87
AS661616	Hechuanmai	Hechuan, Sichuan	SWAS	494	103.92	30.57	156.02	23.38	1.62	30.67	2.4	11.3	10.38	145.97
AS661617	Huayangxiaomai	Shuangliu, Sichuan	SWAS	494	103.92	30.57	159.51	23.57	1.6	21.69	1.97	11.1	13.09	146.05
AS661618	Wumangmai	Shuangliu, Sichuan	SWAS	504	103.48	30.42	164.58	23.59	1.92	28.21	1.67	12.38	10.89	152.35
AS661619	Huanghuaxiaomai	Qionglai, Sichuan	SWAS	457	103.82	30.42	164.26	20.61	2.03	27.65	2.11	10.4	10.92	145.07
AS661620	Guangguangtou	Xinjin, Sichuan	SWAS	448	104.43	30.85	161.72	23.49	1.64	25.93	2.11	10.63	12.09	139.83
AS661621	Danaoke	Jintang, Sichuan	SWAS	150	107.70	29.85	160.77	19.16	1.66	22	1.82	10.49	10.8	146.67
AS661622	Yangmai	Fengdong, Sichuan	SWAS	664	108.77	28.83	162.36	23.74	1.64	22.82	2.26	12.27	11.99	145.5
AS661625	Hongkejiang	Youyang, Sichuan	SWAS	350	108.98	28.45	156.34	23.66	1.75	24.1	2.26	11.43	10.12	142.08
AS661626	Baikejiang	Xiushan, Sichuan	SWAS	30	115.13	37.05	164.58	24.41	1.47	30.1	1.38	13.21	11.6	152.98
AS661627	Bazhouxiaomai	Yunyang, Sichuan	SWAS	214	109.63	31.40	162.68	24.79	1.8	22.76	2.26	8.94	14.25	142.25
AS661629	Honghuamai	Wuxi, Sichuan	SWAS	209	106.45	29.83	162.04	24.84	1.88	25.69	2.26	11.56	11.12	143.7
AS661630	Chikezhuomai	Beibei, Sichuan	SWAS	209	106.45	29.83	162.36	21.4	1.47	24.02	1.82	8.72	11.63	139.67
AS661631	Beibeixiaomai	Beibei, Sichuan	SWAS	200	106.52	29.38	162.36	23.97	1.34	20.47	1.97	9.5	11.31	132.1
AS661633	Huoshaomai	Baxian, Sichuan	SWAS	2450	101.97	30.05	160.46	23.49	1.52	26.99	1.97	9.62	10.7	137.49
AS661634	Hongmangmaizi	Yidong, Sichuan	Q&T	275	106.10	30.78	157.6	21.89	1.58	30.99	2.26	10.46	10.15	141.24
AS661636	Dongmai	Nanchong, Sichuan	SWAS	275	106.10	30.78	157.29	19.99	1.75	23.86	1.97	10.12	11.02	138.98
AS661637	Zaodongmai	Nanchong, Sichuan	SWAS	233	106.63	30.47	155.7	22.55	1.79	26.17	2.11	9.71	10.5	141.38
AS661639	Hongkekemaizi	Guangan, Sichuan	SWAS	320	106.42	31.03	159.82	24.17	1.52	22.63	1.97	7.76	10.1	130
AS661640	Heshangmai	Pengan, Sichuan	SWAS	761	111.77	37.13	158.55	19.77	1.43	23.13	1.53	9.25	9.76	136.16
AS661641	Hongpimai	Wusheng, Sichuan	SWAS	326	105.88	30.98	158.87	22.2	1.47	24.58	2.11	9.38	13.06	139.81
AS661642	Yicuomao	Xichong, Sichuan	SWAS	326	105.88	30.98	155.38	26.9	1.67	30.49	2.26	11.26	10.15	135.48
AS661643	Baixiaomai	Xichong, Sichuan	SWAS	363	105.97	31.58	160.14	25.3	1.84	30.91	1.82	10.46	9.47	139.25
AS661644	Fangmai	Langzhong, Sichuan	SWAS	363	105.97	31.58	153.17	20.18	1.84	21.67	2.4	8.98	13.59	127.6
AS661645	Baidongmai	Langzhong, Sichuan	SWAS	287	104.62	28.77	162.36	23.46	1.71	20.95	2.11	10.48	14.51	135.51
AS661647	Laolaihong	Yibin, Sichuan	SWAS	587	102.98	29.98	154.75	19.48	1.77	17.97	2.11	8.49	11.31	106.78
AS661648	Baihuamai	Yaan, Sichuan	SWAS	328	106.55	31.07	150.63	21.82	1.99	26.69	2.4	8.81	8.28	118.66
AS661652	Sanyuehuang	Yingshan, Sichuan	SWAS	424	104.13	30.00	161.72	21.05	1.71	24.69	1.53	10.85	11.38	145.65
AS661654	Honghuaxuxumai	Renshou, Sichuan	SWAS	328	103.80	29.18	156.65	18.57	1.64	19.89	1.97	9.57	8.99	118.52
AS661655	Baimaidong	Jianwei, Sichuan	SWAS	1120	113.37	37.07	154.75	20.21	1.88	32.08	1.53	13.14	9.44	151.24
AS661656	Hongkebaidongmai	Neijiang, Sichuan	SWAS	350	105.90	29.37	157.29	23.34	1.86	23.28	2.26	10.27	11.3	136.83
AS661657	Yuqiumai	Yongchuan, Sichuan	SWAS	453	107.80	30.68	157.29	19.34	1.66	26.99	1.82	8.93	10.54	149.22
AS661658	Baikexuxusanyuehuang	Liangping, Sichuan	SWAS	453	107.85	31.08	159.19	21.36	1.54	24.67	2.26	10.32	12.22	138.85
AS661661	Paidengmai	Kaijiang, Sichuan	SWAS	1368	105.47	25.12	154.12	18.92	1.73	18.82	2.11	10.85	11.57	114.22
AS661662	Yumai	Anlong, Guizhou	SWAS	1250	106.60	26.85	157.6	21.01	1.71	29.23	1.97	12.6	10.18	143.7
AS661663	Gaoganhongmai	Xiuwen, Guizhou	SWAS	1235	106.97	27.07	155.07	22.42	2.08	21.67	2.26	11.94	8.57	112.93
AS661664	Dahongmai	Kaiyang, Guizhou	SWAS	760	107.52	26.27	157.6	19.98	1.58	31.95	1.38	13.59	9.99	145.86
AS661665	Yuqiumai	Dongyun, Guizhou	SWAS	1085	106.98	26.45	160.14	19.69	1.69	32.01	1.38	13.49	9.73	149.97
AS661666	Xizhanmai	Longli, Guizhou	SWAS	667	107.88	28.52	159.51	20.41	1.73	27.97	1.67	12.74	10.34	149.56
AS661667	Caoxiepian	Wuchuan, Guizhou	SWAS	723	113.63	37.80	155.38	25.67	1.66	28.93	2.26	13.79	9.28	133.32
AS661668	Huimai	Wuchuan, Guizhou	SWAS	1240	104.90	25.08	149.36	26.51	2.08	14.71	2.4	11.99	8.87	99.81
AS661669	Youmai	Xingyi, Guizhou	SWAS	-	105.77	26.05	156.34	18.53	1.79	21.45	1.67	12.25	10.7	122.13
AS661670	Huanghuamai	Zhenning, Guizhou	SWAS	1235	106.97	27.07	161.41	23.45	1.67	25.84	2.26	11.49	11.47	139.22
AS661671	Guangtoumai	Kaiyang, Guizhou	SWAS	310	109.18	26.67	156.97	18.72	1.49	22.21	1.67	9.01	10.83	141.32
AS661675	Hongmangmai	Jinping, Guizhou	SWAS	540	108.12	28.25	161.41	20.1	1.54	22.89	1.09	10.58	9.92	134
AS661676	Liulengmai	Dejiang, Guizhou	SWAS	580	107.88	27.23	161.09	18.48	1.6	23.67	1.24	7.96	10.76	141.59
AS661678	Baihuamai	Yuqing, Guizhou	SWAS	1080	100.12	24.43	159.19	18.21	1.62	27.58	1.09	9.95	11.05	150.96
AS661679	Tiekemai	Yunxian, Yunnan	SWAS	1587	99.90	24.60	168.38	20.55	1.99	30.62	2.26	13.46	12.15	153.45
AS661681	Fengqingxiaomai	Fengqing, Yunnan	SWAS	1534	103.15	24.90	169.02	22.84	1.92	26.43	2.11	12.09	11.8	140.84
AS661682	Zhushimai	Yiliang, Yunnan	SWAS	850	111.80	36.85	158.87	19.36	1.67	28.1	2.26	9.71	9.92	143.87
AS661683	Zimai	Guangnan, Yunnan	SWAS	1534	103.15	24.90	162.04	20.41	1.54	22.84	2.26	11.27	11.96	148.82
AS661684	Yangmaizi	Yiliang, Yunnan	SWAS	1324	112.75	40.08	154.75	22.98	1.67	30.97	1.82	12.65	10.25	146.91
AS661692	Dahongmai	Zuoyun, Shanxi	NS	1320	113.33	40.10	164.58	21.94	1.54	18.89	2.26	12.04	11.31	138.69
AS661693	Xiaobaimai	Datong, Shanxi	NS	-	107.12	40.88	170.6	22.94	1.73	22.43	2.26	13.13	14.09	135.69
AS661694	Dongdaomei	Hangjinhouqi, Neimenggugu	NS	-	111.17	40.30	154.12	18.73	1.71	28.41	2.11	10.84	13.15	126.67
AS661695	Xiaobaipi	Tuoketuoxian, Neimenggu	NS	239	126.97	47.43	161.72	18.53	1.56	22.43	2.26	12.34	10.78	136
AS661696	Huomaizi	Hailun, Heilongjiang	NES	125	126.55	45.72	164.89	23.74	1.84	25.43	2.26	12.49	12.28	146.67
AS661697	Youmangxiaomai	Jiang, Heilong	NES	280	130.28	45.30	164.89	22.87	1.8	26.47	2.69	12.5	11.6	148.96
AS661698	Hongmang5Hao	Linkou, Heilongjiang	NES	140	127.42	46.12	166.8	26.05	1.82	28.32	2.55	13.32	12.25	148.42
AS661699	Suliandaye	Bayan, Heilongjiang	NES	930	111.13	37.53	153.48	26.51	1.71	25.84	1.97	13.98	14.31	138.53
AS661700	Tutoumai	Nancun, Heilongjiang	NES	3	120.95	31.38	165.85	20.83	1.56	21.19	1.67	11.44	11.8	124.9
AS661701	Changsihong	Kunshan, Jiangsu	YTS	2	119.80	33.78	158.55	18.09	1.47	28.84	2.11	10.72	11.09	157.89
AS661703	Baiyuhua	Funing, Jiangsu	YTS	4	120.62	31.30	155.7	19.92	1.71	25.69	1.53	12.62	9.63	152.19
AS661704	Jianzitou	Wuxian, Jiangsu	YTS	5	120.73	31.67	159.82	17.85	1.56	28.04	1.97	9.12	10.34	152.41
AS661705	Nanjinghuang	Changshu, Jiangsu	YTS	4	119.40	32.20	161.41	21.25	1.47	25.45	1.53	10.14	11.83	140.36
AS661706	Zhenjiang3-96	Zhenjiang, Jiangsu	YTS	8	120.68	28.15	155.38	18.4	1.77	25.82	1.24	12.42	9.5	154.54
AS661707	Baidongheshangmai	Yongjia, Zhejiang	YTS	8	120.68	28.15	157.92	19.64	1.62	20.26	1.82	9.06	11.38	128.81
AS661709	Yongheguangtoumai	Yongjia, Zhejiang	YTS	30	121.42	29.28	160.46	21.15	1.62	24.52	2.11	8.34	11.31	139.35
AS661710	Guangtou	Ninghai, Zhejiang	YTS	5	121.15	30.05	160.46	17.81	1.56	16.82	1.82	9.24	9.28	125.63
AS661711	Huangpi	Yuyao, Zhejiang	YTS	24	116.65	39.90	164.89	19.13	1.41	24.47	2.26	12.83	10.57	154.75
AS661712	Shangxuezao	Fuqing, Fujian	SAS	39	117.60	24.17	148.73	26.13	2.08	29.12	2.84	14.05	8.66	128.94
AS661713	Guwanchisui	Zhangpu, Fujian	SAS	15	119.63	27.10	157.29	20.25	2.05	26.36	1.97	13.08	9.83	144.31
AS661715	Heshangmai	Fuan, Fujian	SAS	124	114.83	25.83	151.58	22.03	1.73	33.84	2.26	11.37	11.93	148.12
AS661716	Guangchangliuyemai	Ganzhou, Jiangxi	SAS	70	117.60	28.42	158.24	21.29	1.86	26.97	1.97	13.47	11.42	157.38
AS661717	Guangtoumai	Hengfeng, Jiangxi	YTS	87	118.23	28.67	156.34	19.41	1.84	28.36	1.24	12.74	10.44	157.72
AS661718	Heshangmai	Yushan, Jiangxi	YTS	52	114.92	28.23	158.87	19.12	1.67	22.23	2.11	10.2	9.34	133.92
AS661720	Shichuan	Shanggao, Jiangxi	YTS	65	119.42	35.98	162.36	20.79	1.54	27.23	2.11	13.32	11.02	151.72
AS661722	Xiaoyouzimai	Zhucheng, Shandong	Y&H	33	115.70	36.83	163.94	18.5	1.84	23.17	1.97	8.15	9.54	120.95
AS661723	Guangzongxiaomai	Linqing, Shandong	Y&H	103	117.95	35.25	163.63	17.87	1.3	22.41	1.09	7.43	11.96	133.13
AS661724	Baisuizi	Feixian, Shandong	Y&H	1100	113.42	36.20	158.24	18.01	1.62	11.87	1.24	9.16	11.05	103.01
AS661725	Yejihong	Qingyun, Shandong	Y&H	52	114.52	33.78	175.36	28.28	1.47	31.73	1.09	10.95	13.83	148.55
AS661726	Baimanghong	Xihua, Henan	Y&H	68	112.75	32.15	161.72	22.46	1.51	21.54	1.53	11.43	10.67	151.17
AS661729	Shanlinguomai	Xiangyang, Hubei	YTS	200	110.82	32.85	156.97	22.08	2.38	17.63	2.4	11.83	10.02	115.85
AS661730	Wuhongcao	Yunxian, Hubei	YTS	68	115.40	30.78	158.55	21.3	1.56	24.06	2.26	12.06	8.81	125.88
AS661732	Changshamai	Luotian, Hubei	YTS	420	109.47	30.28	161.41	20.97	1.41	29.14	1.67	12.94	9.83	154.58
AS661734	Baimayaxiaomai	Dongshi, Hubei	YTS	30	114.38	30.68	152.21	28.23	2.23	22.84	2.84	12.15	9.31	120.66
AS661735	Baimai	Hubei	YTS	330	111.25	31.87	159.51	15.46	1.95	13.89	1.97	10.33	10.92	89.65
AS661736	Baimaizi	Baokang, Hubei	YTS	110	112.75	32.15	156.65	24.53	2.38	19.39	2.26	12.12	9.83	121.74
AS661737	Dongmai	Zaoyang, Hubei	YTS	75	111.33	30.77	149.36	21.99	1.69	23.65	2.69	13.48	9.52	113.07

Supplemental Table S1 Information of the 707 wheat accessions assessed in the present study.

Accession No.	Landrace name	Origin	Geographic distribution[†]	Atitude (m)	East longitude (°)	Northern Latitude (°)	BLUP of 8 agronomic traits[‡]							
							FD	FLL	FLW	PDL	SH	SL	TN	PH
AS661738	Niqiuchuan	Yichang, Hubei	YTS	900	112.92	35.78	162.04	19.7	1.49	20.6	1.38	11.68	11.38	147.05
AS661739	Zhuganqing	Yunxian, Hubei	YTS	29	115.20	29.83	155.38	18.76	1.94	27.04	1.24	12.61	10.44	155.19
AS661740	Guanyuhong	Yangxin, Hubei	YTS	450	109.68	32.32	159.82	17.91	1.45	23.54	1.24	11.79	10.34	135.55
AS661742	Hongmang	Zhuxi, Hubei	YTS	330	111.25	31.87	156.34	17.84	1.75	21.93	1.53	10.52	8.02	126.12
AS661744	Hongmangzi	Baokang, Hubei	YTS	20	115.65	30.20	167.43	19.71	1.56	19.17	1.67	9.34	13.09	133.53
AS661745	Hongxu	Guangji, Hubei	YTS	93	111.78	30.83	162.04	18.33	1.86	25.47	1.97	10.41	9.92	151.38
AS661746	Xiaoganmai	Dangyang, Hubei	YTS	307	109.68	32.32	160.77	20.78	1.52	25.41	2.26	13.96	12.31	155.9
AS661747	Caoxieban	Zhushan, Hubei	YTS	45	115.42	30.23	158.55	15.82	1.47	21.08	2.11	10.12	10.8	145.7
AS661748	Peishanbai	Qichun, Hubei	YTS	60	114.87	30.45	161.09	18.49	1.62	21.47	1.82	10.92	10.18	138.93
AS661749	Yangmai	Huanggang, Hubei	YTS	110	112.75	32.15	155.07	21.52	2.18	22.95	1.82	12.17	9.46	104.64
AS661750	Yuanzhui	Zaoyang, Hubei	YTS	900	112.92	35.78	150.95	28.84	2.2	10.56	1.67	13.24	7.58	92.58
AS661751	Tiezimai	Xianning, Hubei	YTS	151	110.68	31.00	155.07	17.43	1.52	22.73	1.97	10.44	13.91	140.58
AS661753	Gaoshanxiaomai	Zigui, Hubei	YTS	307	109.68	32.32	159.51	18.95	1.43	25.97	1.97	12.78	10.57	153.57
AS661754	Quanmang	Zhushan, Hubei	YTS	330	111.25	31.87	160.14	21.05	1.8	28.04	1.09	11.57	9.6	152.83
AS661755	Quanmangxiaomai	Baokang, Hubei	YTS	47	115.17	30.45	162.68	16.48	1.47	22.04	1.24	10.21	10.12	141.62
AS661756	Xishuibagutao	Xishui, Hubei	YTS	68	115.40	30.78	156.97	21.99	1.56	29.34	1.53	12.28	10.57	151.84
AS661757	Zimai	Luotian, Hubei	YTS	200	110.82	32.85	156.65	19.69	1.54	26.39	1.67	11.64	11.57	147.48
AS661758	Pushanba	Yunxian, Hubei	YTS	58	114.00	29.50	166.48	17.37	1.84	21.5	1.67	9.86	10.99	142.97
AS661759	Wugongmai	Chongyang, Hubei	YTS	-	113.38	31.72	155.07	19.06	1.45	21.43	1.38	10.72	12.22	140.66
AS661760	Wugongxu	Suixian, Hubei	YTS	58	114.00	29.50	151.26	22.77	1.84	22.95	1.97	12.83	11.89	124.94
AS661761	Jiyubao	Chongyang, Hubei	YTS	447	111.67	36.25	160.14	18.94	1.51	17.71	1.82	7.08	10.73	123.8
AS661762	Baikeheshangmai	Dongkou, Hunan	YTS	5	116.98	23.68	159.82	19.84	1.36	27.8	1.67	10.76	9.99	149.05
AS661763	Guangtoumai	Raoping, Guangdong	SAS	-	122.03	46.07	149.36	21.1	1.95	28.32	2.69	10.76	9.6	125.93
AS661764	Bendixiaomai	Xingan, Guangxi	SAS	3000	98.83	31.22	161.09	18.46	1.51	26.86	1.97	11.19	10.34	145.14
AS661768	Baiyuxiaomai	Baiyu, Sichuan	Q&T	255	106.97	30.25	173.77	23.14	1.73	17.08	2.26	13.45	13.25	132.03
AS661771	Tuodongmai	Quxian, Sichuan	SWAS	1680	102.57	26.63	162.68	19.63	1.69	23.91	1.38	10.1	10.18	131.24
AS661773	Guangtouxiaomai	Huidong, Sichuan	SWAS	270	107.77	29.32	162.68	26.86	1.82	25.52	2.26	11.79	12.12	142.7
AS661774	Guangtouxiaomai	Wulong, Sichuan	SWAS	3080	100.32	30.93	157.29	23.06	1.62	26.04	2.55	10.46	8.86	142.11
AS661775	Gaoshanzaoshuxiaomai	Xinlong, Sichuan	Q&T	2640	101.02	30.03	169.02	23.74	1.49	14.91	2.11	13.84	13.38	113.31
AS661777	Zhuonixiaomai	Yajiang, Sichuan	SWAS	4200	98.10	32.98	174.72	22.16	1.82	19.82	2.26	12.46	16.32	131.34
AS661778	Ranriwumangmai	Shiqu, Sichuan	Q&T	447	111.67	36.25	162.36	21.92	1.69	18.6	2.26	11.63	16.87	114.71
AS661779	Qianqianmai	Guanling, Guizhou	SWAS	1247	106.47	26.57	166.8	22.79	1.56	25.91	1.82	11.25	13.65	146.26
AS661780	Wuhuaxiaomai	Qingzhen, Guizhou	SWAS	1587	99.90	24.60	160.46	24.82	2.78	18.82	1.67	14.83	10.8	126.2
AS661781	Daheimai	Fengqing, Yunnan	SWAS	1700	99.60	24.83	163.63	20.89	2.31	22.36	2.26	9.12	10.6	129.29
AS661782	Guangyiwuyuemai-8	Changning, Yunnan	SWAS	1580	99.25	24.03	174.09	20.18	1.75	25.65	1.97	8.76	18.4	152.02
AS661783	Wumulongxiaobaimai	Yongde, Yunnan	SWAS	1684	102.43	23.37	163.94	19.09	1.47	26.69	2.11	9.43	11.05	136.15
AS661784	Bendixiaomai	Honghe, Yunnan	SWAS	1889	102.58	24.60	155.7	19.85	1.79	22.91	2.4	9.63	10.15	135.09
AS661785	Bendiyoumangxiaomai	Jinning, Yunnan	SWAS	1434	103.43	24.40	160.14	19.33	1.38	23.54	2.26	11.79	10.63	133.23
AS661786	Bendizimai	Mile, Yunnan	SWAS	1587	99.90	24.60	161.09	23.32	1.66	25.1	1.97	13.24	12.7	145.01
AS661790	Youmanghuakemai	Fengqing, Yunnan	SWAS	2340	99.28	27.17	161.41	16.03	1.97	25.21	2.26	9.96	9.7	136.63
AS661791	Guangtoubaikemai	Weixi, Yunnan	SWAS	447	111.67	36.25	172.82	20.12	1.58	23.86	1.97	11.36	12.25	140.95
AS661792	Guangtoumai	Simao, Yunnan	SWAS	913	100.72	23.33	165.85	23.26	2.01	28.95	2.11	13.03	11.54	143.65
AS661793	Guangtoumai-2	Jinggu, Yunnan	SWAS	1587	99.90	24.60	162.36	20.05	1.88	26.49	2.4	11.18	11.44	132.93
AS661794	Hongganguangtoumai	Fengqing, Yunnan	SWAS	1715	100.30	25.22	162.36	22.66	1.8	26.08	2.26	11.39	10.8	146.67
AS661795	Huakemai	Weishan, Yunnan	SWAS	1587	99.90	24.60	168.06	19.89	1.52	26.08	1.82	10.47	12.86	139.45
AS661796	Ruankemai	Fengqing, Yunnan	SWAS	1684	102.50	25.22	162.68	18.94	1.82	25.97	1.82	9.46	11.41	140.78
AS661798	Chunmai	Fumin, Yunnan	SWAS	1123	109.50	35.82	150.63	27.21	1.77	26.65	2.26	9.49	12.64	115.98
AS661800	Sanyuehuang	Luochuan, Shanxi	NW	460	109.35	32.38	161.41	19.67	1.3	23.13	1.24	10.98	12.31	129.09
AS661803	Xiaosanyuehuang	Pingli, Shanxi	YTS	419	108.60	34.10	156.65	18.68	1.73	16.56	2.26	10.81	10.25	113.46
AS661804	Xiaohongmai	Huxian, Shanxi	NW	934	110.13	34.08	160.77	20.96	1.79	17.84	2.26	7.21	10.67	119.62
AS661806	Shanyangxue	Luonan, Shanxi	NW	-	112.25	36.17	162.04	19.2	1.28	23.84	1.09	10.18	10.6	137.73
AS661807	Makouxi	Zhenan, Shanxi	YTS	705	109.95	33.92	163.31	17.25	1.56	20.5	1.82	8.73	11.6	147.55
AS661809	Changmanghong	Shangxian, Shanxi	Y&H	-	108.03	35.03	160.77	21.51	1.64	24.95	1.09	12.06	9.25	152
AS661810	Changsuimai	Binxian, Shanxi	NW	570	110.30	33.67	163.63	19.52	1.36	23.45	1.09	9.34	9.21	127.1
AS661812	Shisanmai	Danfeng, Shanxi	Y&H	820	109.00	33.67	162.68	16.94	1.58	15.02	1.67	9.31	12.44	147.4
AS661814	Bailanmai	Zuoshui, Shanxi	Y&H	705	109.95	33.92	161.09	17.79	1.41	19.89	1.09	8.66	12.28	130.29
AS661815	Baiximai	Shangxian, Shanxi	Y&H	820	107.98	33.53	176.94	18.91	1.38	20.45	1.09	9.19	14.83	134.82
AS661817	Baiyudongmai	Foping, Shanxi	Y&H	684	109.87	33.52	174.09	20.05	1.51	23.3	1.09	11.5	13.96	139.57
AS661819	Baiyanglazi	Shanyang, Shanxi	Y&H	320	109.37	32.85	162.68	16.71	1.71	18.04	1.53	7.71	10.41	140.82
AS661822	Baiyanglazi	Xunyang, Shanxi	SWAS	470	110.85	33.50	167.11	15.21	1.66	19.63	1.24	8.26	11.93	143.92
AS661823	Baiwugongcao	Shangnan, Shanxi	Y&H	590	110.83	35.42	162.99	17	1.81	22.6	1.38	11.18	13.67	151.01
AS661824	Baijiantiao	Sanyuan, Shanxi	NW	1123	109.50	35.82	163.94	19.07	1.34	26.04	1.09	8.74	15.82	140.76
AS661825	Baisuihonglibeidimai	Luochuan, Shanxi	NW	642	106.13	33.33	172.19	20.83	1.1	26.23	1.09	9.53	13.35	130.92
AS661827	Lanhuamai	Zhenan, Shanxi	YTS	450	108.90	32.30	164.26	18.07	1.45	21.5	1.38	7.62	12.89	131.09
AS661831	Laoxianmai	Langao, Shanxi	YTS	934	110.13	34.08	158.87	19.69	1.86	24.19	1.53	9.4	11.7	136.67
AS661832	Laomanmai	Luonan, Shanxi	NW	934	110.13	34.08	162.68	14.33	1.32	17.37	1.09	7.59	10.41	144.78
AS661833	Huitoucao	Luonan, Shanxi	NW	423	108.78	34.60	158.55	15.39	1.38	22.39	1.09	9.57	10.47	143.9
AS661837	Hongdatou	Sanyuan, Shanxi	NW	320	109.37	32.85	162.04	18.09	1.56	23.36	1.09	10.16	13.6	148.53
AS661839	Honghuamai	Xunyang, Shanxi	SWAS	934	110.13	34.08	170.28	20.93	1.41	17.56	1.09	11.61	10.54	133.75
AS661842	Hongxingliu	Xunyang, Shanxi	SWAS	450	108.90	32.30	166.48	19.62	1.47	23.08	2.11	11.32	15.67	146.28
AS661843	Hongxumai	Langao, Shanxi	YTS	320	109.37	32.85	162.36	19.41	1.49	23.04	1.09	11.79	10.86	131.51
AS661845	Yugongdiao	Xunyang, Shanxi	SWAS	250	109.03	32.72	157.92	19.53	1.52	26.34	2.26	11.32	8.44	131.03
AS661846	Shanxibai	Ankang, Shanxi	YTS	510	108.93	34.15	169.02	20.68	1.56	17.06	1.24	10.71	14.35	140.92
AS661847	Yangxiaomai	Changan, Shanxi	NW	800	106.25	32.83	156.97	18.24	1.32	21.41	1.09	8.65	10.08	128.96
AS661848	Canlaomai	Ningqiang, Shanxi	SWAS	934	110.13	34.08	170.92	20.58	1.71	15.87	1.53	11.49	15.22	134.25
AS661850	Cantiaomai	Luonan, Shanxi	NW	684	109.87	33.52	165.85	18.86	1.6	22.49	1.24	10.68	12.86	155.6
AS661851	Hulutou	Shanyang, Shanxi	Y&H	322	110.12	32.82	162.99	18.02	1.73	23.99	1.24	8.54	10.92	146.45
AS661853	Pushanba	Baihe, Shanxi	YTS	820	109.00	33.67	169.33	23.19	1.47	18.78	1.67	11.09	12.93	134.53
AS661855	Deguolan	Zuoshui, Shanxi	Y&H	39	116.60	40.13	163.63	20.36	1.88	24.65	1.97	10.98	11.25	124.23
AS661856	Dabaimai	Yongdeng, Gansu	NWS	1454	100.17	39.15	169.97	19.21	1.64	14.06	2.11	9.66	14.09	122.73
AS661860	Lanzhouhongmaizi	Yongchang, Gansu	NWS	1756	101.08	38.80	167.43	26.02	2.53	16.13	2.26	14.68	13.28	121.36
AS661861	Hongmaomai	Shandan, Gansu	NWS	1756	101.08	38.80	164.26	19.72	1.64	20.97	2.26	11.91	10.34	135.22
AS661864	Ziganzi	Shandan, Gansu	NWS	2215	101.43	36.03	177.89	24.85	1.69	18.93	2.26	7.37	12.75	129.67
AS661865	Daliuyuehuang	Guide, Qinghai	Q&T	2124	102.10	36.50	172.82	20.14	1.66	16.63	2.26	10.87	12.57	119.46
AS661866	Dahongmai	Pingan, Qinghai	Q&T	2747	100.25	38.20	164.89	27.84	2.07	21.56	2.26	14.9	10.34	131.05
AS661867	Xiaohongmai(Babao)	Qilian, Qinghai	Q&T	-	98.10	36.30	160.77	20.13	1.54	18.39	1.67	10.97	14.57	120.97
AS661868	Xiaohongmai	Donglan, Qinghai	Q&T	2215	101.43	36.03	168.38	21.33	1.77	18.52	0.95	11.72	10.92	127.59
AS661871	Maohongmai	Guide, Qinghai	Q&T	1990	102.50	36.50	165.85	20.12	1.77	16.48	2.26	11.1	12.89	123.01
AS661872	Galaohan	Ledong, Qinghai	Q&T	403	110.72	35.15	163.63	19.58	1.66	15.56	2.26	11.74	10.92	116.96
AS661873	Lanmai	Donglan, Qinghai	Q&T	-	115.22	37.92	166.48	19.07	1.32	16.91	1.97	10.28	10.96	126.48
AS661880	Dabailing(Pingyuan40)	Yuanshulu, Hebei	Y&H	23	115.90	37.78	162.36	18.76	1.26	19.76	1.09	9.89	15.19	135.8
AS661881	Xiaohongmang	Wuyi, Hebei	Y&H	-	115.22	37.92	164.58	19.97	1.39	22.49	1.09	10.77	14.62	140.23
AS661882	Shulu2Hao	Yuanshulu, Hebei	Y&H	10	116.92	38.33	164.89	21.16	1.41	24.49	1.09	11.31	10.57	134.72
AS661885	Shenxianmiao	Cangxian, Hebei	Y&H	-	111.25	35.83	167.11	19.82	1.36	24.3	1.09	9.65	12.41	139.36
AS661886	Biyumai	Yuanfencheng, Shanxi	NW	111	128.47	49.58	162.36	17.91	1.52	27.41	1.09	10.48	9.76	137.73
AS661887	Dangdizhong	Xunke, Heilongjiang	NES	140	127.42	46.12	172.19	23.5	1.66	23.78	2.26	13.29	12.7	143.44
AS661888	Yanghuomai	Bayan, Heilongjiang	NES	1160	105.72	34.58	169.65	23.79	1.52	25.97	2.26	12.62	13.47	141.7
AS661891	Baimai	Tianshui, Gansu	SWAS	403	110.72	35.15	162.36	17.44	1.17	23.41	1.09	8.72	11.47	135.17
AS661892	Dingqingmaoyingmai	Changdong, Xizang	Q&T	-	94.38	29.57	168.7	16.26	1.66	16.8	2.26	8.4	12.44	100.9
AS661895	Wangkamai	Chaya, Xizang	Q&T	-	92.60	29.15	169.97	15.93	1.67	14.28	2.69	11.32	16.72	118.27
AS661896	Wumangmaoyingmai	Jiacha, Xizang	Q&T	-	91.78	29.85	179.16	21.83	1.79	20	2.55	12	13.15	120.37
AS661897	Wumangbaimai	Mozhugongka, Xizang	Q&T	-	97.18	31.15	169.65	17.45	1.54	18.54	2.4	12.8	13.38	127.22
AS661900	Zharenmabu	Changdong, Xizang	Q&T	-	93.18	29.07	169.02	20.52	1.69	15.37	2.26	11.83	10.94	111.14
AS661901	Zharenzhuoma	Langxian, Xizang	Q&T	-	91.95	27.98	168.38	21.56	1.66	18.26	2.4	10.85	12.15	132.15
AS661903	Zhaguogouqumai	Cuona, Xizang	Q&T	-	92.20	29.05	175.99	19.42	1.82	21.04	2.26	10.26	11.8	132.56
AS661904	Zhana	Qusong, Xizang	Q&T	-	98.60	29.68	173.45	21.75	1.36	19.5	2.26	12.98	14.9	123.95
AS661908	Zhongmangzhuoma	Mangkang, Xizang	Q&T	38	116.95	36.68	160.14	25.59	2.01	27.78	2.84	10.64	8.87	137.86
AS661910	Maoyingmai	Cuona, Xizang	Q&T	-	97.62	30.62	175.67	18.6	1.67	22.04	2.26	12.67	13.73	138.48
AS661911	Changmangmaoyingmai	Chaya, Xizang	Q&T	-	97.62	30.62	163.94	21.67	1.95	20	2.26	12.64	12.02	121.72
AS661912	Changmangbailimai	Chaya, Xizang	Q&T	-	90.68	30.02	152.53	22.77	1.75	28.12	2.4	12.46	9.79	126.39
AS661914	Changmangpeizhuo	Donglongdeqing, Xizang	Q&T	-	89.67	29.33	176.31	20.35	1.66	16.8	2.55	10.01	13.83	116.52
AS661915	Renbuchun	Renbu, Xizang	Q&T	-	97.62	30.62	169.97	20.26	1.51	19.67	2.26	10.83	15.06	127.24
AS661916	Rendachangguangmai	Chaya, Xizang	Q&T	-	92.60	29.15	172.19	16.75	1.69	13.19	2.26	8.5	13.93	100.5
AS661919	Pizhuo	Jiacha, Xizang	Q&T	-	91.78	29.22	172.82	20.72	1.73	18.52	2.69	11.77	11.8	123.73
AS661921	Dongmenhongtuzi	Naidong, Xizang	Q&T	-	91.78	29.22	182.33	19.02	1.47	13.13	1.38	11.32	13.35	124.88
AS661922	Dongmentutoumai	Naidong, Xizang	Q&T	-	92.60	29.15	169.97	20.06	1.75	15.89	2.26	8.07	12.76	112.4
AS661923	Donglaichanghongmai	Jiacha, Xizang	Q&T	-	120.32	37.62	177.26	20.72	1.73	23.04	2.26	9.18	12.99	135.36
AS661925	Baixiaomai	Linzhi, Xizang	Q&T	-	97.18	31.15	169.02	24.96	1.66	17.11	1.24	9.56	12.86	133.7
AS661928	Jiachangmai	Changdong, Xizang	Q&T	-	92.60	29.15	162.36	17.42	1.71	17.26	1.97	8.8	11.28	121.41
AS661929	Jiachazharenzhuoma	Jiacha, Xizang	Q&T	-	92.60	29.15	152.85	21.02	1.66	26.3	2.55	12.63	11.63	127.12
AS661931	Jiachadongchunmai	Jiacha, Xizang	Q&T	-	92.60	29.15	172.19	20.37	1.82	20.5	2.26	11.88	12.96	130.32
AS661932	Jiachahongmai	Jiacha, Xizang	Q&T	-	92.60	29.15	180.43	21.05	1.86	17.93	1.97	11.2	14.86	128.93
AS661933	Jiachahongying	Jiacha, Xizang	Q&T	-	92.60	29.15	171.55	18.93	1.75	16.43	2.55	8.9	12.01	131.27
AS661934	Jiachashumai	Jiacha, Xizang	Q&T	-	88.17	29.38	174.09	23.1	1.56	20.95	2.84	14.55	10.6	133.54
AS661936	Jidingwumangchunmai	Xietongmen, Xizang	Q&T	-	91.33	29.25	-	-	-	-	-	-	-	-
AS661938	Jilinchunmai	Zhanang, Xizang	Q&T	-	90.87	29.27	162.04	25.24	1.77	29.21	2.26	11.66	11.38	120.33
AS661939	Jixiongzhaga	Gongga, Xizang	Q&T	-	117.80	34.77	160.14	19.9	1.58	25.89	1.53	11.61	11.76	138.26
AS661941	Dazizaxiaomai-3	Dazi, Xizang	Q&T	-	87.68	29.05	152.21	20.64	1.94	15.3	2.84	12.6	9.73	107.2

Supplemental Table S1 Information of the 707 wheat accessions assessed in the present study.

Accession No.	Landrace name	Origin	Geographic distribution†	Atitude (m)	East longitude (°)	Northern Latitude (°)	BLUP of 8 agronomic traits‡							
							FD	FLL	FLW	PDL	SH	SL	TN	PH
AS661945	Quxiajizhuo	Lazi, Xizang	Q&T	-	91.12	29.68	169.33	20.31	1.75	15.95	1.67	9.76	15.15	115.08
AS661948	Quhongmai	Lasachengguanqu, Xizang	Q&T	-	97.62	30.62	175.36	19.97	1.84	17.63	2.26	11.24	10.21	123.56
AS661951	Tuanjiejizhuo	Chaya, Xizang	Q&T	-	92.47	28.43	169.33	18.57	1.82	14.17	2.11	9.36	14.09	119.93
AS661955	Xingrongchunmai	Longzi, Xizang	Q&T	-	96.97	30.05	158.24	21.63	1.69	19.8	2.26	9.93	10.86	130.05
AS661956	Hongxiaomai	Basu, Xizang	Q&T	-	91.78	29.22	165.53	17.25	1.62	18.6	2.26	11.56	15.09	114.7
AS661957	Hongheshangtou	Naidong, Xizang	Q&T	-	96.97	30.05	173.14	19.05	1.62	19.19	2.55	12.4	15.96	132.41
AS661961	Suzhuozhuoma	Basu, Xizang	Q&T	-	88.93	27.48	164.26	20.22	1.84	18.84	2.4	12.44	12.31	134.9
AS661962	Gangjijizhuo	Yadong, Xizang	Q&T	-	95.63	29.90	159.51	17.92	1.79	27.43	2.26	11.4	11.28	136.29
AS661963	Tumaomai	Bomi, Xizang	Q&T	201	117.85	36.48	174.09	19.37	1.8	18.47	2.11	9.31	10.86	126.42
AS661964	Shagangchun	Kangma, Xizang	Q&T	-	92.60	29.15	-	-	-	-	-	-	-	-
AS661966	Longnanchangmang	Jiacha, Xizang	Q&T	-	94.38	29.57	172.19	19.61	1.51	19.13	2.26	10.97	10.25	133.15
AS661967	Linzhizaxiaomai	Linzhi, Xizang	Q&T	-	94.05	29.20	159.19	20.55	2.1	20.87	2.26	8.86	11.09	126
AS661969	Jieguozharenbusu	Milin, Xizang	Q&T	-	91.95	27.98	174.09	17.11	1.88	21.13	2.11	10.86	12.18	134.16
AS661970	Nianzhadongxiaomai	Cuona, Xizang	Q&T	-	97.62	30.62	169.33	19.37	1.79	18.15	2.26	7.27	11.67	139.55
AS661975	Xuelongzhuo	Chaya, Xizang	Q&T	-	95.63	29.90	170.6	18.87	1.39	13.5	2.26	8.16	15.54	97.98
AS661976	Bomizamai-5	Bomi, Xizang	Q&T	-	91.78	29.22	155.38	19.11	1.69	27.28	1.67	11.19	10.6	128.26
AS661977	Zedangmaoying	Naidong, Xizang	Q&T	-	91.78	29.22	152.53	19.24	1.95	18.41	2.55	9.41	13.46	108.37
AS661978	Zedangzaxiaomai	Naidong, Xizang	Q&T	-	97.62	30.62	169.65	18.7	1.67	16.08	2.26	9.87	13.28	112.07
AS661979	Zongshamai	Chaya, Xizang	Q&T	26	120.45	36.38	169.65	16.86	1.77	10.87	2.11	11.19	12.28	118.94
AS661980	Dingrichangmang	Dingri, Xizang	Q&T	-	87.80	28.35	153.8	19.86	1.52	22.84	1.53	9.94	10.83	125.27
AS661981	Dingjiechun	Dingjie, Xizang	Q&T	-	89.05	29.67	163.31	20.28	1.49	20.43	1.97	10.99	11.05	142.27
AS661983	Chunxiaomai	Nanmulin, Xizang	Q&T	-	85.97	28.17	155.38	22.53	2.18	24.23	2.11	12.23	10.7	119.59
AS661986	Nielamuwumang	Nielamu, Xizang	Q&T	-	93.18	29.07	163.94	16.32	1.64	16.87	1.38	9.28	12.89	103.78
AS661988	Langxiangongzhuo	Langxian, Xizang	Q&T	-	94.38	29.57	166.48	17.51	1.47	18.11	1.38	6.87	13.38	117.48
AS661995	Lulangbaimai	Linzhi, Xizang	Q&T	-	91.95	27.98	165.53	20.74	1.47	16.3	2.11	11.2	11.8	122.14
AS661997	Cuonabaimai	Cuona, Xizang	Q&T	-	94.05	29.20	176.31	18.89	1.66	18.41	2.26	13.01	13.12	125.41
AS661998	Deyangzamai	Milin, Xizang	Q&T	-	91.12	29.68	170.28	21.76	1.56	23.97	1.53	10.04	11.54	123.73
AS662001	Cangmai-169	Lasa, Xizang	Q&T	-	87.17	29.23	157.29	20.83	1.51	25.58	1.97	10.38	10.96	126.79
AS662003	Wumangxiaomai	Angren, Xizang	Q&T	26	120.45	36.38	159.82	19.33	1.62	20.63	2.26	9.55	16.25	111.4
AS662005	Baiyingwumangxiaomai	Jiangzi, Xizang	Q&T	-	87.07	28.62	166.16	19.01	1.64	18.45	2.26	11.12	13.52	112.24
AS662007	Quxiaxiaomai	Dingri, Xizang	Q&T	-	87.17	29.23	146.51	17.98	1.38	22.13	2.11	8.61	10.41	112.3
AS662010	Zhuntangyuzhuo	Angren, Xizang	Q&T	40	116.57	34.68	155.07	22.21	1.8	20.34	1.97	11.57	11.02	125.58
AS662012	Xiaohongmang	Fengxian, Jiangsu	Y&H	34	117.23	34.27	166.48	19.95	1.39	24.69	1.09	9.67	11.57	137.56
AS662013	Honghulutoubaimai	Xuzhou, Jiangsu	Y&H	920	110.45	31.10	166.16	17.32	1.41	21.15	1.09	9.38	14.02	130.09
AS662014	Sanyuehuang	Shennongjia, Hubei	YTS	307	109.68	32.32	150.63	22.18	2.07	22.91	2.4	10.41	8.47	110.06
AS662016	Hongmanglijianmai	Zhushan, Hubei	YTS	450	109.68	32.32	166.16	18.13	1.71	22.63	1.09	9.19	12.18	152.48
AS662017	Heshangtou	Zhuxi, Hubei	YTS	450	109.68	32.32	156.34	23.64	2.05	21.8	2.26	12.12	10.12	118.76
AS662018	Nanjingzao	Zhuxi, Hubei	YTS	307	109.68	32.32	152.53	23.73	2.12	24.52	2.11	11.11	9.25	107.8
AS662019	Tiegancao	Zhushan, Hubei	YTS	15	122.40	37.17	152.47	19.79	1.8	21.36	2.11	11.25	8.83	113.91
AS662020	Molengmai	Ninglang, Yunnan	SWAS	1483	104.32	24.98	152.85	21.42	1.97	25.39	2.69	11.84	9.96	123.02
AS662021	Dongmangmai	Luoping, Yunnan	SWAS	-	97.18	31.15	159.19	19.11	1.75	22.45	1.67	9.85	10.13	136.18
AS662022	Shenggexiaomai	Changdong, Xizang	Q&T	-	88.93	27.48	163.94	19.21	1.77	20.3	2.26	11.74	13.93	134.66
AS662026	Xiakangbu	Yadong, Xizang	Q&T	-	87.07	28.62	163.94	22.22	1.82	16.41	2.26	12.93	11.63	121.6
AS662028	Zhaxigangzhuo	Dingri, Xizang	Q&T	-	94.38	29.57	153.48	16.31	1.51	23.3	1.24	10.66	11.73	115.32
AS662030	Baimadianzhuo	Linzhi, Xizang	Q&T	-	94.38	29.57	172.82	18.19	1.3	22.47	1.67	11.59	12.86	136.14
AS662031	Jirizhuo	Linzhi, Xizang	Q&T	-	91.78	29.85	175.04	18.52	1.73	19.89	2.26	13.3	12.15	137.68
AS662033	Jigupizhuo	Mozhugongka, Xizang	Q&T	-	97.62	30.62	-	-	-	-	-	-	-	-
AS662034	Dadangzhuo	Chaya, Xizang	Q&T	-	91.12	29.68	173.14	22.27	1.58	13.93	2.26	13.46	14.48	113.42
AS662036	Yangridasui	Lasa, Xizang	Q&T	54	116.00	39.70	177.89	20.06	1.56	17.8	2.55	9.46	11.8	126.37
AS662037	Jiangdangtutou	Naidong, Xizang	Q&T	-	97.48	28.65	166.16	20.98	1.94	20.41	2.26	10.35	12.25	136.12
AS662038	Gongjizhuo	Chayu, Xizang	Q&T	-	97.48	28.65	158.87	23.81	1.71	27.6	2.11	14.98	9.76	130.12
AS662039	Shamabizhuo	Chayu, Xizang	Q&T	-	94.38	29.57	169.02	19.41	1.6	18.13	2.11	8.49	12.96	141.75
AS662041	Layuedasui	Linzhi, Xizang	Q&T	-	95.63	29.90	173.77	22.74	1.79	27.28	1.24	13.7	13.33	138.96
AS662042	Yigongzhuo	Bomi, Xizang	Q&T	-	95.63	29.90	173.77	19.96	1.6	21.23	2.26	10.05	15.9	142.36
AS662043	Bomidongqu	Bomi, Xizang	Q&T	-	97.48	28.65	160.46	18.21	1.43	28.08	1.97	11.5	10.41	151.87
AS662046	Sangjiubizhuo	Chayu, Xizang	Q&T	-	91.37	29.67	174.72	21.21	1.79	17.93	1.09	9.82	13.59	125.83
AS662049	Tajizhuo	Dazi, Xizang	Q&T	-	89.67	29.33	170.28	22.99	1.71	15.28	2.26	9.55	15.51	113.37
AS662050	Pumanghongmai	Pumang, Xizang	Q&T	-	90.08	29.48	169.65	21.79	1.62	15.41	2.26	12.44	11.63	119.65
AS662052	Tarongzhuo	Nimu, Xizang	Q&T	42	122.03	37.18	158.87	18.14	1.64	22.47	2.4	10.42	11.6	136.65
AS662053	Fanshenzhuo	Qiongjie, Xizang	Q&T	1739	104.63	35.00	172.5	21	2.07	17.26	2.26	12.31	9.96	118.69
AS662055	Huomaizi	Longxi, Gansu	NWS	1420	105.18	34.18	160.77	15.98	1.39	13.28	1.09	9.48	10.41	107.2
AS662056	Baiyumai	Lixian, Gansu	SWAS	1567	105.28	34.00	161.41	18.39	1.47	15.02	1.24	8.49	13.51	137.17
AS662057	Baizaomai	Xihe, Gansu	SWAS	1495	104.88	34.73	169.97	21.56	1.51	23.67	1.09	11.65	19.85	143.27
AS662059	Laobaimai	Wushan, Gansu	NWS	1420	105.18	34.18	166.16	22	1.64	22.26	2.26	11.73	12.28	140.47
AS662061	Youmangyangmai	Lixian, Gansu	SWAS	957	105.72	33.75	162.04	18.82	1.64	20.17	1.24	7.67	11.93	127.69
AS662067	Huomai	Lingwu, Ningxia	NWS	1183	105.67	37.48	163.94	21.93	1.79	24.32	2.26	11.6	11.7	143
AS662068	Hongtuzi	Huinong, Ningxia	NWS	10	121.60	37.40	169.33	20.4	1.54	17.63	2.26	13.02	14.15	132.64

† Geographic distribution of the tested landrace accessions. The ten Chinese agro-ecological zones includes: NW (Northern Winter Wheat Zone), Y&H (Yellow and Huai River Valleys Facultative Wheat Zone), YTS (Middle and Low Yangtze Valleys Autumn-Sown Spring Wheat Zone), SAS (Southern Autumn-Sown Spring Wheat Zone), SWAS (Southwestern Autumn-Sown Spring Wheat Zone), NES (Northeastern Spring Wheat Zone), NS (Northern Spring Wheat Zone), NWS (Northwestern Spring Wheat Zone), Q&T (Qinghai-Tibetan Plateau Spring-Winter Wheat Zone), and XJ (Xinjiang Winter-Spring Wheat Zone).

‡ BLUP of 8 agronomic traits were obtained from a previous report (Liu, Y., Lin, Y., Gao, S., Li, Z., Ma, J., and Deng, M., et al. (2017). A genome-wide association study of 23 agronomic traits in Chinese wheat landraces. Plant J. 91:861–873.).
"-", data unavailable
