# Supplementary material for: A Genome-Wide Association Study of Coleoptile Length in Different Chinese Wheat Landraces
Source: Front Plant Sci. 2020 Jun 4;11:677. doi: 10.3389/fpls.2020.00677 (PMC7287122; doi:10.3389/fpls.2020.00677)
Supplement: Supplementary file 2 [file Data_Sheet_2.PDF]

Supplemental Table S2 The information of high-resolution melt (HRM) marker.

Marker name	Forward primer (5’-3’)	Reverse primer (5’-3’)	Sequence information of production
HRM	GACGCTAGTTGCATCATCT	GCGACTGGTGCTACTATG	GACGCTAGTTGCATCATCTGCCCGCGGGCGAGATCTGCAGGTTGCAGCGCC[A/G]TTGTCATTGCATAGTAGCACCAGTCGC
