# Supplementary material for: A Genome-Wide Association Study of Coleoptile Length in Different Chinese Wheat Landraces
Source: Front Plant Sci. 2020 Jun 4;11:677. doi: 10.3389/fpls.2020.00677 (PMC7287122; doi:10.3389/fpls.2020.00677)
Supplement: Supplementary file 3 [file Data_Sheet_3.PDF]

Supplemental Table S3 List of candidate genes located on the genomic region of *QCl.sicau-6B.2* (Chr. 6B: 508.17–509.26Mb).

Gene Name	Chromosom	Position	Homologous Gene of Arabidopsi	Definition	Homologous Gene of Rice	Definition
TraesCS6B01G282000	6B	508.18	*AT1G72230*	Cupredoxin superfamily protein	*osa:4330178*	Blue copper protein
TraesCS6B01G282100	6B	508.54	*AT4G17550/ G3Pp4*	Major facilitator superfamily protein (Kawai *et al* ., 2014)	*osa:4330176*	Putative glycerol-3-phosphate transporter 4
TraesCS6B01G282200	6B	508.61	*AT4G17560*	Ribosomal protein L19 family protein	*osa:9272428*	50S ribosomal protein L19-2, chloroplastic
TraesCS6B01G282300	6B	508.61	*AT5G47180*	Plant VAMP (vesicle-associated membrane protein) family protein	*osa:4349306*	Vesicle-associated protein 2-1
TraesCS6B01G282400	6B	508.82	*AT2G44770*	ELMO/CED-12 family protein	*osa:4330174*	ELMO domain-containing protein A
TraesCS6B01G282500	6B	508.94	*AT2G44745/ WRKY12*	WRKY family transcription factor (Wang *et al.* , 2010; Sanchez *et al.* , 2012; Li *et al*	*osa:4330173*	Probable WRKY transcription factor 12
TraesCS6B01G282600	6B	509.26	*AT2G44740/ CYCP4;1*	Cyclin p4;1 (Torres Acosta *et al.,* 2004)	*osa:4330172/ P0491E01.9*	Cyclin-P4-1-like (Kikuchi et al. 2003)
TraesCS6B01G530800LC	6B	508.20				
TraesCS6B01G530900LC	6B	508.60				
TraesCS6B01G531000LC	6B	508.60			*osa:107279392*	Uncharacterized LOC107279392
TraesCS6B01G531100LC	6B	508.60	*ArthMp029*	Hypothetical protein	*osa:107279669*	Uncharacterized LOC107279669
TraesCS6B01G531200LC	6B	508.60	*AT2G34320*	Polynucleotidyl transferase, ribonuclease H-like superfamily protein	*osa:6450185/ orf258 (OrsajM_p08*	Orf258 (Hiratsuka et al. 1989)
TraesCS6B01G531300LC	6B	508.64	*ArthCp015/ petN*	Cytochrome b6/f complex subunit VIII	*osa:3131455/ycf6*	Cytochrome b6/f complex subunit N (Boeckmann et al. 2003; Hiratsuka et al. 1989)
TraesCS6B01G531400LC	6B	508.82	*AT5G35695*	Nuclease HARBI1-like protein	*osa:107280916*	Protein ALP1-like
TraesCS6B01G531500LC	6B	508.83	*AT1G43722*	Nuclease	*osa:107278809*	Uncharacterized LOC107278809
