# Supplementary material for: A Genome-Wide Association Study of Coleoptile Length in Different Chinese Wheat Landraces
Source: Front Plant Sci. 2020 Jun 4;11:677. doi: 10.3389/fpls.2020.00677 (PMC7287122; doi:10.3389/fpls.2020.00677)
Supplement: Supplementary file 4 [file Data_Sheet_4.PDF]

Supplemental Table S4 QTL/markers for CL identified in previous studies.

Gene / QTL name	Marker/Marker interval	Reported Chromosome	IWGSC Reference Sequence			Reference
			Chromosome	Star	End	
QTL-1B	XpGTG-mTCGA294	1B				Yu and Bai, 2010
QTL-3D	XpCATG-mGCG449	3D				
QTL-4D-1	XRht-D1	4DS				
QTL-4D-2	Xgwm194	4DL				
QTL-5AS	XpCAT-mGAC306	5AS				
QTL-5B	XpACT-mCTA113	5B				
qCL.3B.1	cfp56–gpw7774	3B				Singh *et al*, 2015
qCL.3B.2	gpw7774–cfb3530	3B				
qCL.3B.3	cfb3059–cfb3375	3B				
qCL.4B.1	gwm368 - wmc125	4B				
qCL.6B.1	gpw1017 - barc79	6B	582.38	582375665(barc 178	582375641	
Rht2		4DS				Li *et al.*, 2016a
Rht1		4BS				
QCL.stars-4DC1	IWA5381	4DC	4D	62469466	62469660	
QCL.stars-4BS1	IWA8564	4BS				
	IWA2963	4BC	4B	482647961	482647761	
QCL.stars-2DC1	IWA8304	2DC				
QCL.stars-2DS1	IWA1799	2DS				
QCL.stars-5BL1	IWA4903	5BL				
	IWA2931	5BL				
QCL.stars-1BS1	IWA2355	1BS				
	IWA1698	UNK				
	IWA2205	UNK				
QCL.stars-4BS2	IWA3798	4BS				
QCL.stars-5B/5D	IWA3580	5BL/5DL				
	XcsME1	4BS				Rebetzke *et al.*, 2001
	XksuC2	4BL				
	P31/M58-2	5AL				
	NW1574	3AS				Spielmeyer *et al.*, 2007
	NW3106	6AS				
	ksuG9c	1AS				Rebetzke *et al.*, 2007
	wPt-0615	2BS				
	Stm55ltgag	2DS				
	wPt-8855	3BS				
	Rht-B1b	4BS				
	psr426	5AL				
	psr326b	5DS				
	wmc76	7BS				
	wPt-6463	7BS				
	stm55ltgag	2DL				
	RGA74.15	5BS				
	wmc474	2BS				
	gwm515c	2DS				
	gwm165	4AS				
	Rht-D1b	4DS				
	barc178	6BL	664.63	664631847	664631827	
	P36/M43-1	2DS				
	abg75c	3BS				
	abg3a	5DS				
	P35/M39-9	6BL				
	KsuH9c	7AL				
	bcd310	1AL				
	P41/M51-2	5DS				
	gwm637	4AS				
	gwm219	6BL	674.84	674843297	674843316	
	wsnp_Ex_c3253_5995011	1AS	1A	62614504	62614304	
	wsnp_Ku_c207_407862	1BS				
	wsnp_CAP11_c2596_1325540	1BL	1B	674498355	674498554	
	wsnp_Ex_c14760_22866930	2BS				
	wsnp_Ra_c17636_26538543	2DL				
	wsnp_Ku_c38911_47455924	3AS				
	wsnp_Ra_c17636_26538543	3D2				
	wsnp_Ex_c13615_21393511	4AS	4A	570267331	570267531	
	wsnp_Ex_c14026_21924297	4BS				
	Rht-D1 (Rht2)	4DS				
	wsnp_Ex_c683_1341113	4DS	4D	54446907	54447107	
	wsnp_Ku_c12562_20256747	5BL				
	wsnp_Ex_c214_421541	5BL	5B	42947783	42947983	

Supplemental Table S4 QTL/markers for CL identified in previous studies.

Gene / QTL name	Marker/Marker interval	Reported Chromosome	IWGSC Reference Sequence			Reference
			Chromosome	Star	End	
	wsnp_Ku_c55961_59662821	5DS	5D	540833502	540833702	
	wsnp_RFL_Contig2182_1514692	6AS				
	wsnp_Ex_rep_c70767_69655253	6BL	6B	692783145	692783345	
	wsnp_Ex_c61603_61581218	7AS				
	wsnp_Ex_c12117_19381493	1AS	1A	49529904	49529704	Rebetzke *et al.*, 2014
	wsnp_Ex_c13310_20984763	1BS	1B	489898413	489898213	
	wsnp_Ex_c8188_13842273	1DL	1D	410464792	410464592	
	wsnp_Ex_c30447_39360584	2BS				
	wsnp_CAP7_c2782_1329707	2DS				
	wsnp_Ku_c38911_47455924	3AS				
	wsnp_Ex_rep_c102478_87635370	3AL				
	wsnp_Ex_rep_c66380_64573939	3BL	3D	606883198	606882998	
	wsnp_Ex_rep_c69093_68002098	4AS				
	wsnp_Ex_c14026_21924297	4BS	4B	38026016	38025909	
	Rht-D1 (Rht2)	4DS				
	wsnp_Ex_c683_1341113	4DS	4D	54446907	54447107	
	wsnp_Ku_c6464_11320381	5BL	5B	402843711	402843511	
	wsnp_Ex_rep_c70951_69806211	6AS				
	wsnp_Ex_rep_c71537_70252046	6BL	6B	690730582	690730716	
	wsnp_Ex_c42653_49180485	7AL	7A	83631404	83631594	
	wsnp_Ex_c43009_49439922	7AL	7A	1720051	1719868	
	wsnp_Ex_c323_629581	7BS	7B	703705316	703705116	
