# Supplementary material for: A Genome-Wide Association Study of Coleoptile Length in Different Chinese Wheat Landraces
Source: Front Plant Sci. 2020 Jun 4;11:677. doi: 10.3389/fpls.2020.00677 (PMC7287122; doi:10.3389/fpls.2020.00677)
Supplement: Supplementary file 5 [file Data_Sheet_5.PDF]

Table S5. List of significant markers associated with eight agronomic traits.

Trait	Marker	Chromosome	Physical Distance (Mb)	$-LOG_{10}^{(P)}$	PVE (%)
SL	A27000	1A	260.94	4.80	2.70
SH	A60113	1A	268.12	5.25	3.00
FD	A24882	1B	47.18	14.54	10.65
FD	A51733	1B	295.94	5.27	3.09
FD	SNP1393	1B	295.94	5.46	3.08
SH	SNP1436	1B	367.43	4.81	2.60
SH	A20367	1B	466.73	6.66	3.81
SL	SNP2641	2A	4.81	4.78	2.68
SL	SNP2650	2A	6.20	4.84	2.72
FLL	A3560	2A	8.02	5.14	3.04
SL	A3824	2A	62.10	4.83	2.86
SL	B4013	2A	157.12	4.93	2.78
SL	A4026	2A	161.57	5.18	3.04
PDL	SNP4096	2B	15.72	4.86	2.72
PDL	SNP4110	2B	17.72	5.12	2.88
PDL	SNP4113	2B	17.96	4.81	2.69
SH	A4990	2B	22.55	4.86	2.88
SH	SNP4918	2B	594.21	5.14	2.81
SH	SNP4932	2B	602.00	4.87	2.64
SH	A6147	2B	730.09	5.28	3.16
FD	A50645	2D	10.47	4.93	3.00
SL	SNP5855	2D	22.16	5.02	2.84
SL	B7250	2D	22.16	5.09	2.90
SL	B7249	2D	22.16	4.95	2.81
SL	A3783	2D	22.17	5.04	2.94
SL	SNP5860	2D	23.75	5.48	3.14
FD	A32552	2D	33.96	15.12	10.25
FLL	A32552	2D	33.96	7.93	5.06
SL	A32552	2D	33.96	5.94	3.57
TN	A32552	2D	33.96	5.79	3.46
PH	A32552	2D	33.96	5.21	3.07
FD	SNP5895	2D	33.96	16.01	10.23
FLL	SNP5895	2D	33.96	8.06	4.87
TN	SNP5895	2D	33.96	6.42	3.73
SL	SNP5895	2D	33.96	5.46	3.13
PH	SNP5895	2D	33.96	4.82	2.68
SL	A31904	2D	424.20	5.48	3.38
SL	SNP6161	2D	600.16	5.62	3.23
FD	A31711	2D	618.33	5.48	3.77
SH	A4627	2D	642.20	4.78	2.75
SH	A7907	2D	647.97	5.52	3.64
SH	A31937	2D	648.06	5.62	3.41
SH	A31595	2D	648.10	5.13	2.98
SH	A6534	2D	648.17	6.20	3.82
SH	A26997	2D	648.47	6.86	4.20
SH	SNP6464	2D	649.11	5.97	3.33
SH	A49528	2D	649.11	7.31	4.65
SH	A48098	2D	649.11	7.12	4.28
SH	SNP6465	2D	649.11	7.42	4.25

Table S5. List of significant markers associated with eight agronomic traits.

Trait	Marker	Chromosome	Physical Distance (Mb)	$-LOG_{10}^{(P)}$	PVE (%)
SH	A31988	2D	649.11	5.75	3.52
SH	A7920	2D	649.12	4.78	2.92
SH	SNP6466	2D	649.12	6.17	3.46
SH	A31979	2D	649.30	5.64	3.27
SH	A7927	2D	649.34	5.79	3.40
SH	SNP6467	2D	649.34	5.78	3.21
SH	A28755	2D	649.39	5.93	3.51
SH	SNP6469	2D	649.62	6.35	3.57
SH	A25217	2D	649.62	6.03	3.62
SH	A31413	2D	649.88	6.38	3.75
SH	A7930	2D	649.88	6.28	3.72
SH	A7931	2D	650.07	6.11	3.65
SH	A50587	2D	650.21	7.75	4.84
SH	A7936	2D	650.21	7.12	4.30
SH	A7959	2D	650.47	4.81	2.88
SH	A32167	2D	650.56	7.29	4.47
SH	A50842	2D	650.68	8.22	5.37
SH	A37913	2D	650.68	8.31	5.36
SH	A51381	2D	650.68	8.22	5.08
SL	SNP6515	3A	8.23	5.08	2.88
FD	A8479	3A	275.00	18.81	13.66
SH	SNP6930	3A	661.48	6.15	3.44
SH	B10623	3A	661.48	6.09	3.79
SH	SNP6931	3A	661.66	8.12	4.70
PDL	A33360	3A	685.54	4.95	2.90
SH	B9117	3A	707.91	4.77	3.04
FD	A34201	3B	3.04	4.83	2.92
FD	A34847	3B	4.76	8.15	5.42
FD	SNP7206	3B	4.76	6.92	4.03
FD	A34395	3B	33.96	4.89	2.79
PDL	A9722	3B	263.24	4.88	2.83
PDL	A9716	3B	304.28	5.01	2.95
SL	A28208	3B	456.96	5.19	2.96
SH	A52251	3B	512.74	4.83	2.78
SH	SNP8047	3B	685.88	5.10	2.78
SH	B10661	3B	685.88	5.35	3.23
SH	A9982	3B	688.16	5.46	3.08
SH	SNP8059	3B	696.33	5.31	2.91
SH	A9998	3B	696.33	5.90	3.43
SH	B10637	3B	696.33	5.48	3.02
SH	B10624	3B	696.33	8.70	5.65
SH	SNP8067	3B	700.76	5.51	3.04
SH	SNP8088	3B	711.24	5.56	3.07
SH	SNP8280	3B	767.06	5.58	3.09
SH	B8620	3D	47.34	4.96	2.72
SH	A8535	3D	392.96	5.24	2.97
SH	SNP8603	3D	392.96	4.87	2.64
TN	A11319	3D	603.46	4.94	2.81
SH	A56583	4A	662.64	5.27	3.31

Table S5. List of significant markers associated with eight agronomic traits.

Trait	Marker	Chromosome	Physical Distance (Mb)	$-LOG_{10}^{(P)}$	PVE (%)
FLW	A37624	4A	703.66	5.70	3.37
SH	A37493	4A	708.61	5.97	3.92
FLW	SNP9577	4A	709.27	5.12	2.84
FLW	SNP9581	4A	709.42	5.88	3.32
FLW	B12724	4A	709.85	4.83	2.78
FLW	SNP9584	4A	709.85	6.95	4.01
FLW	SNP9601	4A	714.15	5.12	2.84
FD	A37679	4A	737.65	5.18	3.01
FD	SNP9704	4A	737.65	5.04	2.82
SH	A38862	4B	65.60	5.27	3.21
SH	A52471	4B	405.91	5.41	2.99
SL	A39252	4B	436.89	4.77	2.69
FD	A66467	4B	459.54	5.08	3.25
FLW	A38218	4B	601.79	4.83	3.11
FLW	A12908	4B	602.40	5.14	3.18
SL	A4030	4D	423.87	5.66	3.48
SL	B13932	5A	36.74	5.03	2.85
SL	SNP10546	5A	36.74	5.03	2.85
SL	SNP10552	5A	38.60	5.44	3.11
SL	SNP10553	5A	38.61	5.19	2.95
SH	B13943	5A	78.42	4.83	2.71
FD	A10438	5A	677.13	5.50	3.43
FLW	A39332	5A	706.05	6.07	3.71
FD	A41422	5B	223.13	5.05	3.12
FLW	A14378	5B	355.45	5.64	3.25
FLW	A14416	5B	417.44	5.76	3.54
SH	A14538	5B	483.22	5.50	3.19
FLL	B15369	5B	487.44	4.92	2.79
FLL	B15370	5B	487.44	4.81	2.72
FLL	SNP11894	5B	487.44	4.92	2.79
FLL	SNP11907	5B	490.40	4.92	2.79
SH	A23941	5B	494.56	4.96	2.73
PDL	A14682	5B	495.63	4.90	2.75
SL	A13791	5B	511.07	6.29	3.67
FLL	A41446	5B	511.61	5.09	3.14
SL	A14730	5B	527.03	4.80	2.86
FLL	A14716	5B	528.06	4.85	3.03
TN	SNP12103	5B	548.68	5.78	3.31
TN	A14887	5B	548.68	5.40	3.37
FD	A2701	5B	573.81	7.05	4.38
FD	A2702	5B	573.81	5.67	3.45
PDL	A15646	5B	685.22	5.04	2.86
FLW	B16333	5B	693.99	5.02	2.83
FLW	SNP12530	5B	693.99	4.78	2.63
FLW	A41248	5B	694.04	5.21	2.91
FD	A15409	5B	711.93	5.00	2.98
FD	SNP12614	5B	711.93	5.28	2.97
FD	A15391	5B	711.93	5.11	3.11
FD	A42265	5D	260.38	4.76	2.89

Table S5. List of significant markers associated with eight agronomic traits.

Trait	Marker	Chromosome	Physical Distance (Mb)	$-LOG_{10}^{(P)}$	PVE (%)
SH	A43005	5D	436.98	5.06	2.94
PDL	B16525	5D	543.47	5.23	3.18
PDL	SNP12800	5D	543.47	5.53	3.15
PDL	A15645	5D	543.50	6.12	3.74
PDL	B16545	5D	543.60	5.60	3.32
PDL	SNP12801	5D	543.60	5.59	3.19
PDL	A42961	5D	545.39	4.91	3.06
PDL	A15656	5D	545.75	5.32	3.28
PDL	SNP12814	5D	546.90	5.53	3.15
PDL	SNP12815	5D	547.03	5.51	3.14
SL	A44705	6A	218.61	5.85	3.39
SL	A23667	6A	232.30	6.58	3.89
SL	A44569	6A	234.02	5.57	3.31
SL	A44571	6A	234.02	5.46	3.26
SL	A27430	6A	310.37	4.82	2.91
SL	A23879	6A	328.65	5.67	3.29
SL	A44092	6A	341.71	4.85	2.76
SL	B17281	6A	358.61	5.57	3.28
SL	B17282	6A	358.61	5.55	3.27
SL	SNP13385	6A	358.61	5.40	3.09
PDL	A43758	6A	360.66	4.79	2.74
SL	SNP13386	6A	361.11	4.78	2.69
PDL	A60769	6A	374.16	5.31	3.03
PDL	A49875	6A	387.19	5.09	2.97
PDL	A16361	6A	387.19	5.71	3.47
SL	A22579	6A	421.53	6.04	4.14
SL	A16407	6A	421.53	6.05	4.16
SL	A16443	6A	453.95	4.83	2.83
SL	A16440	6A	453.95	4.82	2.75
SL	SNP13441	6A	453.95	4.80	2.70
SL	B17314	6A	453.95	4.77	2.71
SL	SNP13445	6A	456.34	4.87	2.74
SL	SNP13446	6A	456.48	5.58	3.20
SL	A60291	6A	485.68	5.15	2.93
FD	A43706	6A	508.37	4.94	2.85
SH	SNP13532	6A	538.47	5.49	3.03
FD	A16825	6B	4.29	5.16	3.00
FD	A16840	6B	4.29	4.89	2.83
PH	A17437	6B	172.58	4.99	2.99
FD	A45349	6B	210.79	5.50	3.47
FD	SNP14520	6B	210.79	4.99	2.78
FD	A45221	6B	543.19	4.85	2.77
PDL	SNP14979	6B	664.46	5.01	2.81
SH	A20872	6B	692.73	5.29	2.98
SL	A17927	6B	700.19	5.15	2.95
SL	A47782	6B	705.21	5.60	3.33
SL	A300	6B	705.23	5.25	3.14
PDL	SNP15502	6D	357.59	5.22	2.95
PDL	B19453	6D	357.59	5.25	2.98

Table S5. List of significant markers associated with eight agronomic traits.

Trait	Marker	Chromosome	Physical Distance (Mb)	$-LOG_{10}^{(P)}$	PVE (%)
PDL	B19454	6D	357.59	4.91	2.76
PDL	SNP15506	6D	379.05	5.56	3.17
FD	A45970	6D	459.05	5.54	3.78
FD	SNP16091	7A	52.03	4.96	2.77
SH	B20272	7A	71.04	5.06	2.98
SH	B20271	7A	71.04	4.80	2.80
SH	B20296	7A	77.43	5.67	3.15
SH	SNP16161	7A	77.43	5.67	3.14
PDL	A50529	7A	89.64	5.13	3.05
SH	A34828	7A	91.72	5.80	3.31
SH	SNP16204	7A	91.72	6.18	3.46
FD	A67434	7A	497.85	7.63	5.08
SH	A19648	7A	631.26	5.63	3.43
PDL	SNP16856	7A	709.27	4.93	2.76
SH	B21023	7A	709.79	4.94	3.02
SH	B21152	7A	712.65	5.90	3.79
SH	A19963	7A	712.81	5.63	3.41
SH	SNP16882	7A	712.81	5.83	3.24
SH	A66331	7A	712.98	5.90	3.46
SH	A19920	7A	713.01	5.02	3.20
SH	B21001	7A	714.34	7.35	4.24
SH	SNP16890	7A	714.34	7.20	4.11
SH	A38600	7A	715.76	7.63	4.65
SH	SNP16902	7A	715.76	7.57	4.35
SH	SNP16909	7A	716.26	4.89	2.65
SH	B21037	7A	718.20	4.81	2.78
SH	A19978	7A	718.31	4.82	2.88
SH	SNP16928	7A	720.13	7.31	4.21
SH	A20034	7A	721.39	7.44	4.60
SH	SNP16963	7A	726.48	5.18	2.84
SH	A20111	7A	731.69	5.54	3.09
SH	A66066	7A	733.36	4.95	2.96
FD	A48014	7B	2.78	6.38	3.69
SH	SNP18168	7B	715.72	4.98	2.71
SH	B21002	7B	715.72	4.89	2.67
SH	A21248	7B	727.38	5.16	2.86
SH	A50550	7B	736.28	4.77	2.69
FD	A65081	7D	31.12	7.80	5.40
SH	A67348	7D	127.73	5.13	3.28
PH	A66077	7D	394.16	4.82	2.76
FLW	SNP18692	7D	556.23	5.03	2.78
